# Collagen IV^α345^ dysfunction in glomerular basement membrane diseases. I. Discovery of a COL4A3 variant in familial Goodpasture’s and Alport diseases

**DOI:** 10.1016/j.jbc.2021.100590

**Published:** 2021-03-26

**Authors:** Elena N. Pokidysheva, Harald Seeger, Vadim Pedchenko, Sergei Chetyrkin, Carsten Bergmann, Dale Abrahamson, Zhao Wei Cui, Eric Delpire, Fernando C. Fervenza, Aaron L. Fidler, Agnes B. Fogo, Ariana Gaspert, Maik Grohmann, Oliver Gross, George Haddad, Raymond C. Harris, Clifford Kashtan, A. Richard Kitching, Johan M. Lorenzen, Stephen McAdoo, Charles D. Pusey, Marten Segelmark, Alicia Simmons, Paul A. Voziyan, Timo Wagner, Rudolf P. Wüthrich, Ming-Hui Zhao, Sergei P. Boudko, Andreas D. Kistler, Billy G. Hudson

**Affiliations:** 1 Division of Nephrology and Hypertension, Department of Medicine, Vanderbilt University Medical Center, Nashville, Tennessee, USA; 2 Center for Matrix Biology, Vanderbilt University Medical Center, Nashville, Tennessee, USA; 3 Nephrology Division, University Hospital Zurich, Zurich, Switzerland; 4 Department of Medicine and Nephrology, University Hospital Freiburg, Freiburg, Germany; 5 Medizinische Genetik Mainz, Limbach Genetics, Mainz, Germany; 6 Department of Anatomy and Cell Biology, University of Kansas Medical Center, Kansas City, Kansas, USA; 7 Renal Division, Peking University First Hospital, Beijing, PR China; 8 Department of Anesthesiology, Vanderbilt University School of Medicine, Nashville, Tennessee, USA; 9 Division of Nephrology and Hypertension, Mayo Clinic, Rochester, Minnesota, USA; 10 Aspirnaut Program, Vanderbilt University Medical Center, Nashville, Tennessee, USA; 11 Department of Pathology, Microbiology and Immunology, Vanderbilt University Medical Center, Nashville, Tennessee, USA; 12 Department of Pathology and Molecular Pathology, University Hospital Zurich, Zurich, Switzerland; 13 Medizinische Genetik Mainz, Limbach Genetics, Mainz, Germany; 14 Clinic of Nephrology and Rheumatology, University Medical Center Goettingen, University of Goettingen, Goettingen, Germany; 15 Division of Pediatric Nephrology, University of Minnesota Medical School and Masonic Children’s Hospital, Minneapolis, Minnesota, USA; 16 Centre for Inflammatory Diseases, Monash University Department Medicine, Nephrology, Monash Health, Clayton, VIC, Australia; 17 Centre for Inflammatory Disease, Imperial College London, London, UK; 18 Department of Biochemistry, Vanderbilt University, Nashville, Tennessee, USA; 19 Department of Internal Medicine, Kantonsspital Frauenfeld, Frauenfeld, Switzerland; 20 Center for Structural Biology, Vanderbilt University, Nashville, Tennessee, USA; 21 Department of Cell and Developmental Biology, Vanderbilt University, Nashville, Tennessee, USA; 22 Vanderbilt-Ingram Cancer Center, Vanderbilt University, Nashville, Tennessee, USA

**Keywords:** collagen, extracellular matrix, diabetic nephropathy, genetic disease, animal model, AS, Alport syndrome, BM, basement membrane, DN, diabetic nephropathy, GBM, glomerular basement membrane, GP, Goodpasture’s disease, PTM, posttranslational modification

## Abstract

Diseases of the glomerular basement membrane (GBM), such as Goodpasture’s disease (GP) and Alport syndrome (AS), are a major cause of chronic kidney failure and an unmet medical need. Collagen IV^α345^ is an important architectural element of the GBM that was discovered in previous research on GP and AS. How this collagen enables GBM to function as a permselective filter and how structural defects cause renal failure remain an enigma. We found a distinctive genetic variant of collagen IV^**α345**^ in both a familial GP case and four AS kindreds that provided insights into these mechanisms. The variant is an 8-residue appendage at the C-terminus of the α3 subunit of the α345 hexamer. A knock-in mouse harboring the variant displayed GBM abnormalities and proteinuria. This pathology phenocopied AS, which pinpointed the α345 hexamer as a focal point in GBM function and dysfunction. Crystallography and assembly studies revealed underlying hexamer mechanisms, as described in Boudko *et al.* and Pedchenko *et al*. Bioactive sites on the hexamer surface were identified where pathogenic pathways of GP and AS converge and, potentially, that of diabetic nephropathy (DN). We conclude that the hexamer functions include signaling and organizing macromolecular complexes, which enable GBM assembly and function. Therapeutic modulation or replacement of α345 hexamer could therefore be a potential treatment for GBM diseases, and this knock-in mouse model is suitable for developing gene therapies.

Diseases of the glomerular basement membrane (GBM) are a major cause of chronic kidney disease, a health problem affecting about 10% of the global population and an unmet medical need (1, 2). Prominent diseases are diabetic nephropathy (DN), Alport syndrome (AS), and Goodpasture’s disease (GP) that are characterized by morphological abnormalities in GBM, ranging from thickening in DN to multilamellations in AS and ruptures due to specific GBM attack by antibodies in GP (3, 4, 5). These abnormalities involve structural alterations in collagen IV, the major GBM component. How collagen IV enables GBM to function as a permselective filter and how its structural alterations cause GBM abnormalities and dysfunction remain an enigma. New insights into these mysteries were revealed in the present study of a distinctive genetic variant of collagen IV, uniquely associated with both AS and GP diseases, coupled with crystallography and animal studies.

Collagen IV is a family of six homologous α-chains, which are distributed within basement membranes (BMs) that underlie the epithelial architecture of the nephron. In pioneering studies of the GBM over 50 years ago, collagen IV was identified as a novel collagen and shown to be structurally altered in DN (6, 7, 8, 9, 10, 11, 12). It was first characterized as a supramolecular network of triple helical protomers composed of α1 and α2 chains (13, 14). In subsequent studies of the GBM in GP and AS, four additional chains were discovered (α3, α4, α5, and α6) (4, 15, 16, 17, 18, 19, 20, 21). The six α-chains coassemble into protomers with three distinct molecular compositions: α121, α345, and α565, which in turn assemble into three distinct supramolecular scaffolds, noted as collagen IV^**α121**^, collagen IV^α345^, and collagen IV^**α556**^ (22, 23). In the nephron, collagen IV^**α121**^ occurs in the GBM, mesangial matrix, Bowman’s capsule BM, and BMs of tubules and capillaries, whereas collagen IV^**α345**^ is restricted to the GBM (24). Moreover, the collagen IV^**α345**^ scaffold is structurally altered in both AS and GP (16, 17, 18, 19, 20, 21), kidney diseases that have served as vanguards for advancing knowledge of structure, function, and dysfunction of the GBM (25), as well as other BMs across the animal kingdom (26).

In GP, an acquired sporadic disorder, autoantibodies target neoepitopes in the α345 NC1 hexamer that connect triple helical protomers in the collagen IV^**α345**^ scaffold of GBM and alveolar BM (4, 27, 28, 29, 30, 31, 32, 33). The autoantibodies alter and break the GBM, causing crescentic injury, manifesting as rapidly progressive glomerulonephritis, often in association with pulmonary hemorrhage, in thousands of people worldwide. The etiology of GP remains unknown but is proposed to be a combination of environmental and genetic factors, eliciting both humoral and cellular autoimmune response (34). Current therapy is limited to plasmaphereses and immunosuppression (35) and, if not initiated at an early disease stage, is often ineffective to prevent end-stage renal failure.

In AS, hundreds of genetic variants are now known in the *COL4A3*, *COL4A4*, and *COL4A5* genes, encoding the three α-chains (36, 37, 38, 39). These pathogenic variants cause GBM abnormalities that underlie a broad array of glomerular phenotypes in millions of people worldwide, ranging from microscopic hematuria to end-stage kidney failure (37, 40). How this plethora of variants causes renal failure remains unknown, which posits a fundamental question “how does the collagen IV^**α345**^ scaffold enable GBM function?” Lack of clarity in answering this question impedes the development of therapies (37). Current treatments have been limited mainly to ACE inhibitors or ARBs that delay progression to renal failure and the need for renal transplant (36, 37, 41).

The development of therapy for these GBM diseases requires knowledge of the pathobiology of the collagen IV^**α345**^ scaffold in relation to structure, assembly, function, and dysfunction. To this end, a variant in the *COL4A3* gene that was found in both a familial GP case and four kindreds of AS provided a unique opportunity to elucidate pathogenic mechanisms of these two GBM diseases.

## Results

### A distinctive variant of α3(IV) collagen (Zurich (Z)-variant) was found in familial GP and Alport patients

#### Clinical histories of two patients with familial GP disease harboring the Z-variant

**Figure 1:**
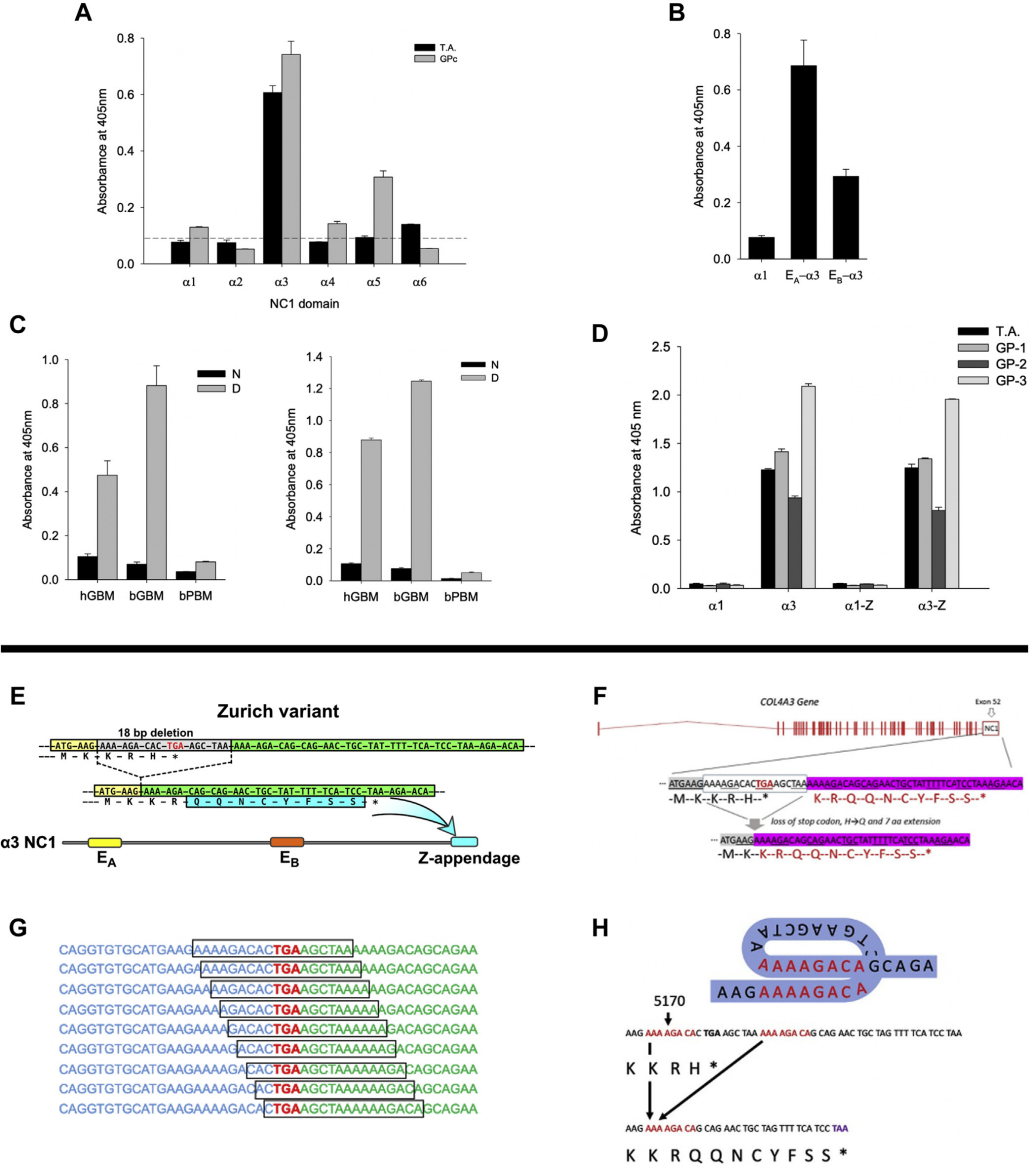
**Immunochemical and genetic analyses of index patient with Zurich variant.***A*, binding of T.A. circulating autoantibodies is restricted to the α3NC1 domain of human collagen IV, while pooled serum from eight sporadic GP cases (GPc) also reacted to α5NC1. Normal human serum does not react to any of NC1 domains as depicted by the dashed line (mean plus 3 × SD). *B*, index patient autoantibodies bind immunodominant epitopes E_A_ and E_B_ of α3NC1, but not the parental α1NC1 domain used to create α1/α3 chimeric proteins bearing E_A_ and E_B_ epitopes. *C*, serum from the index patient reacts with denatured (D), but not native (N) NC1 hexamers purified from human (hGBM) and bovine (bGBM) glomerular basement membrane (*left panel*). This pattern is identical to the reactivity of pooled serum of eight sporadic GP patients (*right panel*). NC1 hexamer from bovine placenta (bPBM), which is composed of GP nonreactive α1 and α2NC1 subunits served as negative control. *D*, the Zurich variant does not directly affect antigenicity of the α3NC1 monomer. Serum from the index patient and from three sporadic GP patients (GP-1, GP-2, GP-3) displayed similar reactivity to the α3NC1 domain and α3NC1 domain variant (α3-Z), but does not react with either α1NC1 domain or α1NC1 chimera (α1-Z) bearing the eight residues extension from the α3NC1 variant of the index patient. These results suggest that Zurich appendage does not represent a neoepitope for patient autoantibodies or affect the presentation of immunodominant E_A_ and E_B_ epitopes in the α3NC1 monomer. All bar graphs with T bars (*A*–*D*) indicate the means and standard errors. *E*, a heterozygous COL4A3 variant (rs765655100: c.5010_∗del(p.His1670_∗167delinsGln∗9)) was discovered by next-generation sequencing in two patients with familial Goodpasture’s disease and designated as Zurich variant. The variant produces a C-terminal modification of the collagen IV α3 chain *via* deletion of 18 base pairs including the stop codon in exon 52 of COL4A3. This results in an alternative stop codon and a consecutive 8-amino acid protein extension named Z-appendage. *F*, the 18 bp in-frame deletion at the C-terminus of NC1 domain introduced an H to Q substitution and added an 8-amino acid appendage to the protein. *G* and *H*, this deletion is flanked by two direct DNA repeats AAAAGACA, suggesting homologous recombination as the mechanism and supporting the possibility of recurrent mutations at this site. Deletions at sites of direct repeats in various genes, including *COL4A3*, have been previously reported and attributed to slippage replication errors (57, 58).

A 24-year-old man (T.A.) presented with pulmonary-renal syndrome. His serum was positive for anti-GBM antibodies at 151 U/ml (reference range: <10 U/ml). The patient required mechanical ventilation and hemodialysis. Whereas pulmonary function completely recovered after treatment with plasmapheresis, glucocorticoids, and cyclophosphamide, the patient remained dialysis-dependent. His mother (A.A.) had suffered fromGP at the age of 45 years also with pulmonary-renal syndrome, resulting in end-stage kidney disease and a transplant. Anti-GBM antibodies at her initial presentation were positive at 72 U/ml, and her native kidney biopsy exhibited severe crescentic glomerulonephritis involving all 23 glomeruli and linear GBM immunofluorescence staining for IgG and complement C3 along the GBM. Given the acute situation with severe crescentic glomerulonephritis, electron microscopy showed no lamellation, thinning, or basket-weaving pattern of the GBM (Fig. S1). She eventually required renal transplantation and currently has severely decreased kidney function. Both patients harbor the HLA allomorph HLA-DR15, known to be a risk factor of GP, and both had normal hearing and ophthalmologic examination. No other family members were affected. Additional clinical details are described in Supplementary Section 1. By indirect ELISA, the autoantibodies from the index patient T.A. displayed similar specificity to autoantibodies from sporadic GP patients with reactivity restricted to the α3NC1 domain and increased binding to denatured GBM NC1 hexamer (Fig. 1, *A*–*C*).

Given that both mother and son had GP disease, a genetic defect was hypothesized. Next-generation sequencing revealed a heterozygous *COL4A3* variant (rs765655100) in both patients located in exon 52, corresponding to the α3NC1 domain (42) (named herein as Zurich variant). The 18-base pair deletion included the stop codon and resulted in the replacement of the last amino acid residue by eight additional amino acids, named herein as the Zurich (Z-) appendage, onto the C-terminal end of the α3NC1 domain (Fig. 1, *E*–*H*, Supplementary Section 2). We assessed whether the Z-appendage itself constituted a neoepitope for GP autoantibodies using serum from the index patient. The appendage, attached to the α3NC1 monomer, did not affect the binding of GP antibodies and did not bind antibodies when attached to a α1NC1 monomer, which is inert to antibody binding (Fig. 1*D*). These results indicate that the Z-appendage does not comprise a neoepitope for GP autoantibodies of the index patient.

**Figure 2:**
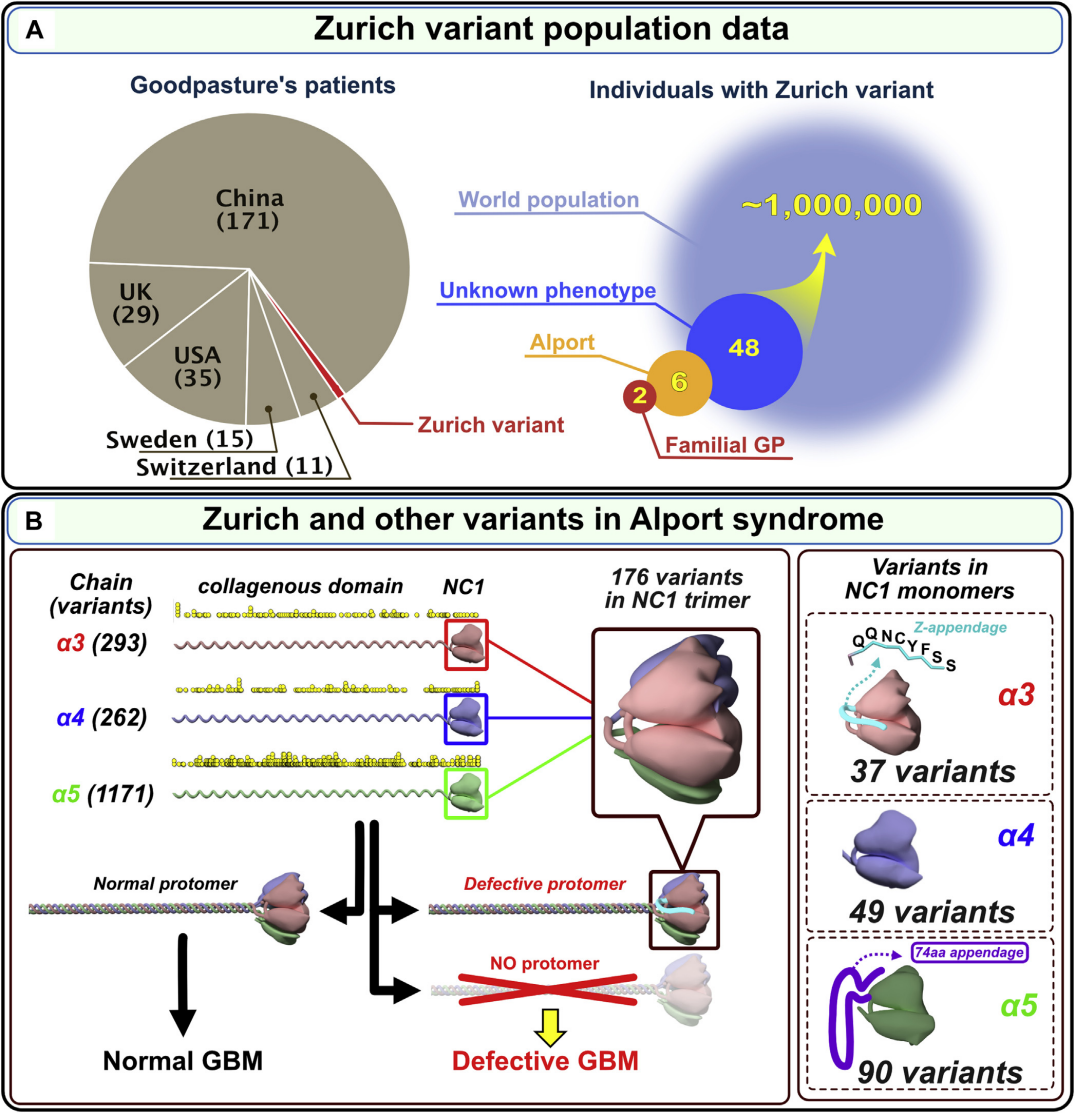
**A genetic variant of α3(IV) collagen (Zurich variant) associated with familial Goodpasture’s disease and Alport syndrome is distinct among the 1700 known Alport variants.***A*, Zurich variant is associated with both familial Goodpasture’s disease (GP) and Alport syndrome. This variant was a risk factor in developing familial Goodpasture’s disease in two Zurich patients (*red pie chart wedge* and *red circle*, n = 2). In several cohorts of GP patients from different countries, the presence of Zurich variant was not detected either by Sanger sequencing of exons 48 to 52 (Switzerland, n = 11; Sweden, n = 15; China, n = 171; and UK, n = 29) or whole exome/genome sequencing (USA, n = 36) as shown in the *gray pie chart*. However, the Zurich variant was detected in patients diagnosed with familial hematuria or suspected Alport syndrome (*tan circle*, n = 6) and in the general population with unknown phenotype (*dark blue circle*, n = 48). The extrapolation of the data set to the world population resulted in estimated one million people carrying the Zurich variant, and thus classified as Alport Syndrome. Therefore, the Zurich variant places them at risk of progressing to renal failure and developing Goodpasture’s disease (*light blue circle*). *B*, the number and location of 1700+ genetic Alport-associated variants (indicated by *yellow dots*, zoom in to see individual dots) in the α3, α4, and α5 chains of collagen IV (*left*). Pathogenic variants cause either loss of α345 protomers from the GBM or assembly of defective α345 protomers that can incorporate into the GBM, causing a broad spectrum of GBM phenotypes. Significant numbers of Alport-associated variants occur within α3, α4, and α5 NC1 domains (*right*). Zurich variant α3NC1 monomer with an 8-amino acid Z-appendage is shown at *top right*. Analogous to Zurich variant, a novel variant α5NC1 monomer with a C-terminal 74-amino acid appendage (59) is shown at *bottom right*. Because these two variants resulted in a C-terminal extension of the protein polypeptide chain, they stood out among over 1700 known variants in the *COL4A3*, *COL4A4*, and *COL4A5* genes associated with Alport syndrome. The Z-appendage incorporated into the collagen IV scaffold of the GBM and served as a pathogenic reporter group that identified a new therapeutic target (*vide infra*).

To determine whether the Zurich variant is present in sporadic GP patients, we performed targeted sequencing of 261 cases from five cohorts, which revealed the absence of the Zurich variant in these patients (Fig. 2*A* and Supplementary Section 3.1). Also, the variant was absent in three patients from a previously reported kindred of familial GP (43) (Supplementary Section 3.2; Fig. S2, Table S1). However, the Zurich variant was found in a general population database (gnomAD; *16* out of ca. 124,000 individuals) of individuals with unknown health profile and in a clinical database (NCBI ClinVar; *32* cases out of ca. 100,000 individuals) (Supplementary Section 3.3). Extrapolation of the gnomAD database resulted in an estimate of about one million people worldwide harboring the Zurich variant (Fig. 2*A*).

#### Clinical histories of patients with Alport syndrome harboring the Z-variant

The Z-variant was also identified in six non-GP individuals with variable renal phenotypes. These individuals were identified in a cohort of patients subjected to targeted Next-Generation Sequencing of *COL4A3*, *COL4A4*, and *COL4A5* genes as part of commercial genetic screening panels (nephrotic syndrome, focal segmental glomerulosclerosis (FSGS), and AS) or evaluation of other suspected genetic renal diseases. In total, *COL4A3* sequencing was performed in 1631 patients: in 311 as part of the nephrotic syndrome panel, in 606 as part of the FSGS panel, in 579 as part of the Alport panel, and in 135, *COL4A3* sequencing was included for other suspected genetic renal diseases. Of note, only a minority of these patients (188) had a histologically confirmed diagnosis. In summary, the rs765655100 Z-variant was identified in a total of six individuals from four families (K, H, U, and S) of diverse origins, with variable renal phenotypes ranging from normal or asymptomatic microhematuria to end-stage kidney disease (Figs. S3 and S4; Table S3). Based on the recently proposed nomenclature of variants in COL4A3, A4, or A5 genes (37), these six patients, described below, were classified as having AS.

In family K (Greek origin), the index patient presented at the age of 23 years with advanced renal failure (creatinine 11.8 mg/dl) and hypertension. By ultrasound, both kidneys were small, consistent with end-stage kidney disease. Therefore, he did not undergo renal biopsy. Proteinuria was 1.9 g/g creatinine at presentation, and he did not have hematuria. Dialysis was initiated shortly after presentation in May 2016. His family history was negative for renal disease including hematuria. Due to sensorineural deafness of the patient as well as his father, an Alport genetic panel was ordered as part of the pretransplant evaluation. Both the patient and his father carried the rs765655100 variant. The father had normal renal function (creatinine 0.9 mg/dl, eGFR 95 ml/min/1.73 m^2^), no proteinuria, no hematuria, and his hearing loss had been previously attributed to job-related noise exposure.

In family H (Arabic origin), the Alport genetic panel was ordered due to microhematuria (50 erythrocytes/μl) in a 10-year-old girl with normal renal function (creatinine 42 μmol/l) and no proteinuria. The girl’s mother also has asymptomatic microhematuria but no proteinuria and normal renal function. Her father suffers from recurrent urolithiasis, but has normal renal function, no proteinuria, and no microhematuria except during symptomatic kidney stone episodes. The rs765655100 variant was found in the daughter and her father (who has no hematuria) but not in the mother (who has microhematuria), while an additional *COL4A4* variant (c.1321_1369+3del, previously associated with familial hematuria/AS) was identified in the daughter and her mother, but not in the father. Neither the index patient nor her parents reported hearing problems.

In family U (mother Caucasian, father Turkish), a 7-year-old girl with normal renal function (creatinine 0.37 mg/dl) and microhematuria (150 erythrocytes/μl) but no proteinuria (urinary protein 86 mg/g creatinine; urinary albumin <20 mg/g creatinine) with a family history of microhematuria in her sister, her mother, and her maternal grandfather was tested by the NGS Alport panel, revealing the heterozygous rs765655100 variant. The index patient and all family members affected by microhematuria have normal hearing.

In family S (Turkish origin), the female index patient showed microhematuria and mild proteinuria but no abnormal albuminuria (urinary protein 260 mg/g creatinine; urinary albumin 7 mg/g creatinine) with normal renal function (creatinine 0.4 mg/dl, eGFR 143 ml/min/1.73 m^2^), first detected at age of 27 years during her second pregnancy. Because her mother reportedly had microhematuria, the index patient was tested with the Alport panel, and the rs765655100 variant was detected without other changes in the tested genes. The index patient and her mother reportedly have normal hearing.

We also performed HLA typing in all patients and family members bearing the rs765655100 *COL4A3* variant, and none of them carried the GP risk HLA allele DRB1∗15:01. Of note, none of the four index patients underwent renal biopsy—either due to advanced chronic renal failure (family K) or due to mild disease. Pedigree trees are presented in Figure. S3 (Supplementary Section 4).

#### Importance of the Z-variant in understanding pathogenesis in GP and AS

About 1700 Alport variants are known in the *COL4A3*, *COL4A4*, and *COL4A5* genes; two are distinct with extensions, termed herein as appendages, onto the C-terminal of the NC1 domains. The Z-variant in the *COL4A3* gene encodes an 8-residue appendage onto the a3NC1 domain and a COL4A5 variant encodes a 74-residue on the α5NC1 domain (Fig. 2*B*). Importantly, the Z-appendage is distinct among the hundreds of variants for several reasons: 1) it occurred in patients with AS and in patients with familial GP disease and therefore, could provide insights into the pathogenesis of both diseases. 2) Its location on the surface of the α345 hexamer places it in juxtaposition with the GP epitopes and T-cell receptor epitope (27, 44, 45, 46), based on a predicted α345 hexamer model (22). The convergence of these pathogenic pathways suggests that a narrow site on the surface of the α345 hexamer is critical in pathogenesis of GP and AS; 3) the Z-appendage is a nontruncating variant, thus can incorporate into the GBM, and 4) its impact on hexamer function may serve as a vanguard for understanding the unknown mechanisms of numerous other variants nearby on the hexamer surface. Collectively, these features prompted us to assess the pathogenicity of the Z-appendage in a mouse model and to elucidate the underpinning biology and chemistry by solving the crystal structure and assembly mechanism of the α345 hexamer.

### Generation of the Zurich variant knock-in mouse

**Figure 3:**
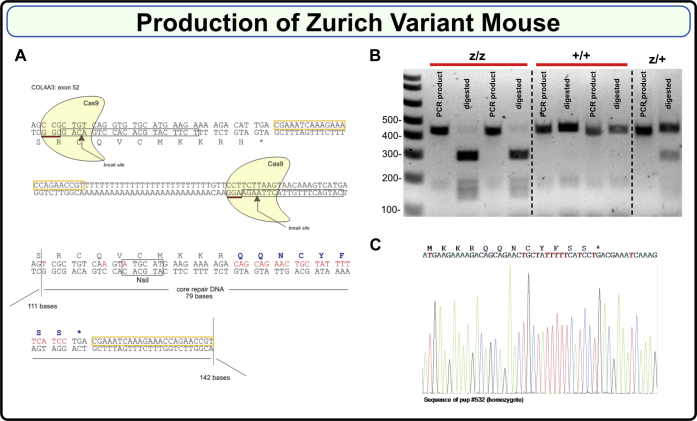
**Production of the Zurich variant mouse.***A*, Zurich variant mouse CRISPR/Cas9 design scheme. Guide RNAs consisting of 20 base pairs each are *boxed*; break sites are indicated. *Lower part* shows repair DNA, which introduces new eight amino acids. *B*, PCR products of 460 bp in wild type (+/+) and 446 bp in the mutant (z/z) were generated using genotyping primers (refer to the Experimental procedures section for the sequences). Mutant PCR product had NsiI restriction site, while wild type did not. PCR products were then digested with NsiI enzyme resulting in appearance of ∼300 bp band for the mutant. Heterozygous (z/+) mice had both undigested and digested bands. *C*, representative Sanger sequencing of gDNA isolated from homozygous F2 Zurich variant mouse.

To assess pathogenicity, we developed a mouse harboring the Zurich variant using CRISPR/Cas9 editing technology. To increase the DNA break efficiency, two overlapping guide RNAs located within exon 52 of the COL4A3 gene were used. Each guide RNA consisted of a 20-base DNA stretch (TCTTCATGCACACCTGACAG and CATGACTTTGTTACTTAAGA, boxed in Fig. 3*A*) directly preceding an NGG as a proto-spacer adjacent motif. A single-strand oligonucleotide repair DNA consisting of a 79 base repair fragment core and flanking 111 and 142 base homology arms was designed (Fig. 3*A*). As shown in Figure 3*A*, mutations were introduced to substitute His1169 with eight new amino acids: QQNCYFSS; disrupt the PAM sequences; introduce a unique NsiI restriction site for genotyping (Fig. 3, *B* and *C*); and eliminate the polyT stretch. Two separate lines were established and backcrossed to C57BL6/J mice for four generations to eliminate possible off target events. Homozygote animals on C57BL/6 background were then generated and line one was expanded and studied. Mice were viable, fertile, and born in the expected Mendelian ratios. For additional details on mouse genotyping, see Supplementary Section 5 and Figure S5.

### Zurich variant phenocopies features of Alport syndrome in a knock-in mouse (Col4a3^z/z^), revealing the α345 hexamer as a focal point in GBM function and dysfunction

**Figure 4:**
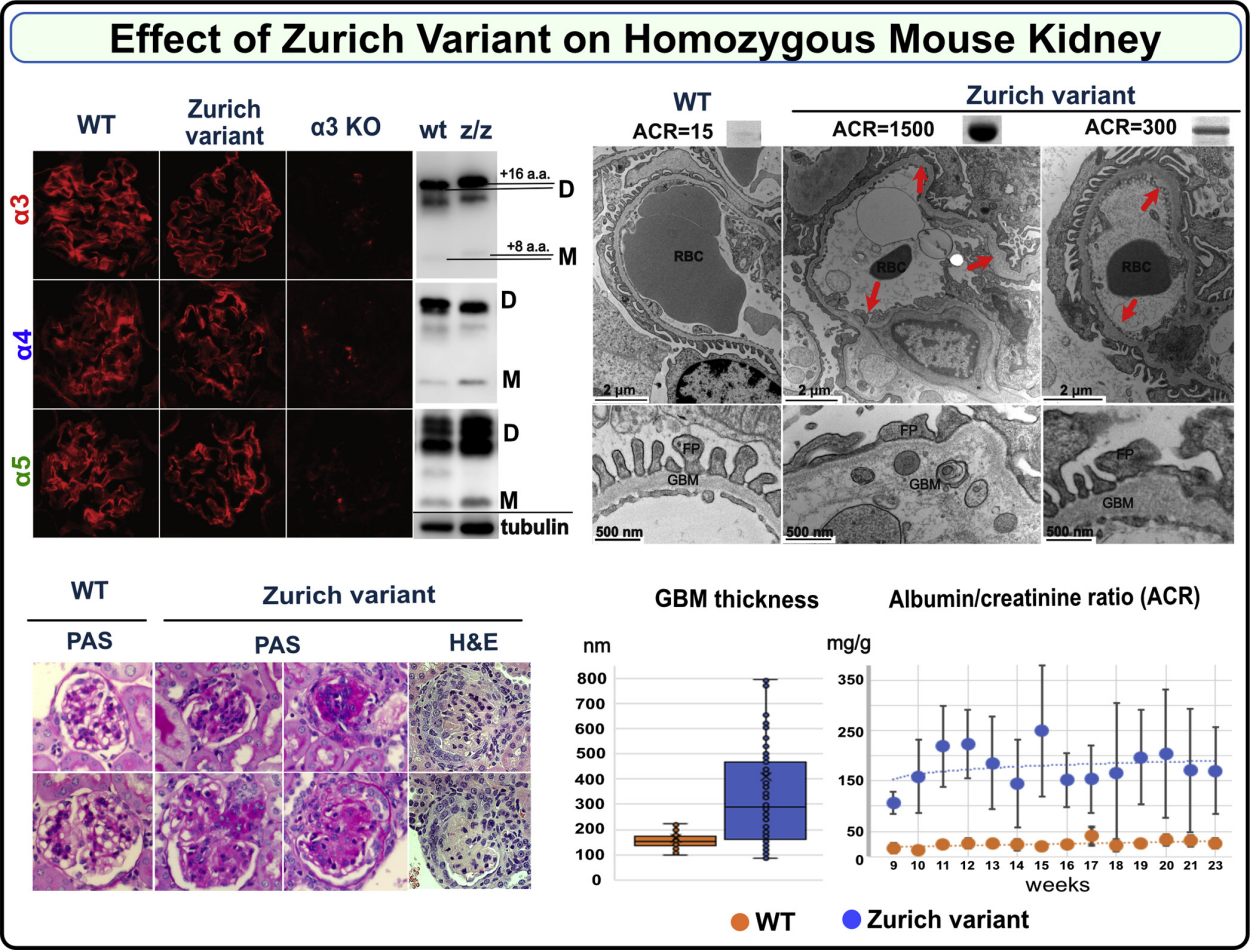
**Zurich variant knock-in homozygous mouse (Col4a3**^**z/z**^**mouse) phenocopies features of Alport syndrome in GBM.** Immunofluorescence staining of renal sections demonstrates deposition of genetically modified α3 chain along with native α4 and α5 chains within collagen IV ^α345^ scaffold in a Col4a3^z/z^ mouse (*top left*). This scaffold did not form in the kidney of Col4a3 KO mouse lacking α3 chain (*top left*). The Zurich variant also did not interfere with hexamer assembly and formation of sulfilimine cross-links as shown by western blot using collagen IV chain-specific antibodies (*top left*, D indicates dimer and M indicates monomer). The shift in the position of mutant α3 bands relative to the control is due to the difference in sequence length. Samples were normalized on kidney weight and tubulin (*bottom* of the western blot). Histological analysis of kidney sections revealed varying degree of glomerular sclerosis in 20% to 70% of the glomeruli in the Col4a3^z/z^ mice (periodic acid–Schiff (PAS)-stained representative glomeruli are shown, magnification 600×) and occasional formation of crescents (*bottom left*, *H&E staining*). Representative transmission electron microscopy (TEM) images of the glomerular capillary loops from two different Col4a3^z/z^ mice and a wild-type control mouse are shown at the *top right*. In Col4a3^z/z^ mice exhibiting high albuminuria (see corresponding urine albumin-to-creatinine ratio (ACR) values and albumin SDS-PAGE bands above the TEM images), the glomerular basement membrane (GBM) was irregularly thinned and thickened, lamellated, and occasionally split with foot process effacement. Animals with moderate albuminuria demonstrated mainly irregular thickening of the GBM (*arrows* and *higher magnification for GBM structure*). GBM thickness (measured from two glomeruli with five images each, from each mouse) was increased in WT (n = 2) *versus* Col4a3^z/z^ (n = 3) mice (*bottom middle*) (*p* < 0.001, Student’s *t* test). Col4a3^z/z^ mice (n = 6) had significantly elevated ACR compared with WT mice (n = 5) (*bottom right*). Both experimental groups included male and female mice.

Homozygous knock-in mice (Col4a3^z/z^) on a C57BL6 background were viable, fertile, and born in the expected Mendelian ratios. The deposition of collagen IV^α345^ scaffold to the GBM assessed by the immunofluorescence was unaffected in the Col4a3^z/z^ mice. Hexamer formation and cross-linking was also unaffected. Direct indication that the Z-appendage incorporated into the scaffold was obtained from the band shift of the α3 NC1 domain derived from Col4a3^z/z^ mouse kidney. (Fig. 4 and Supplementary Section 6; Fig. S6).

**Figure 5:**
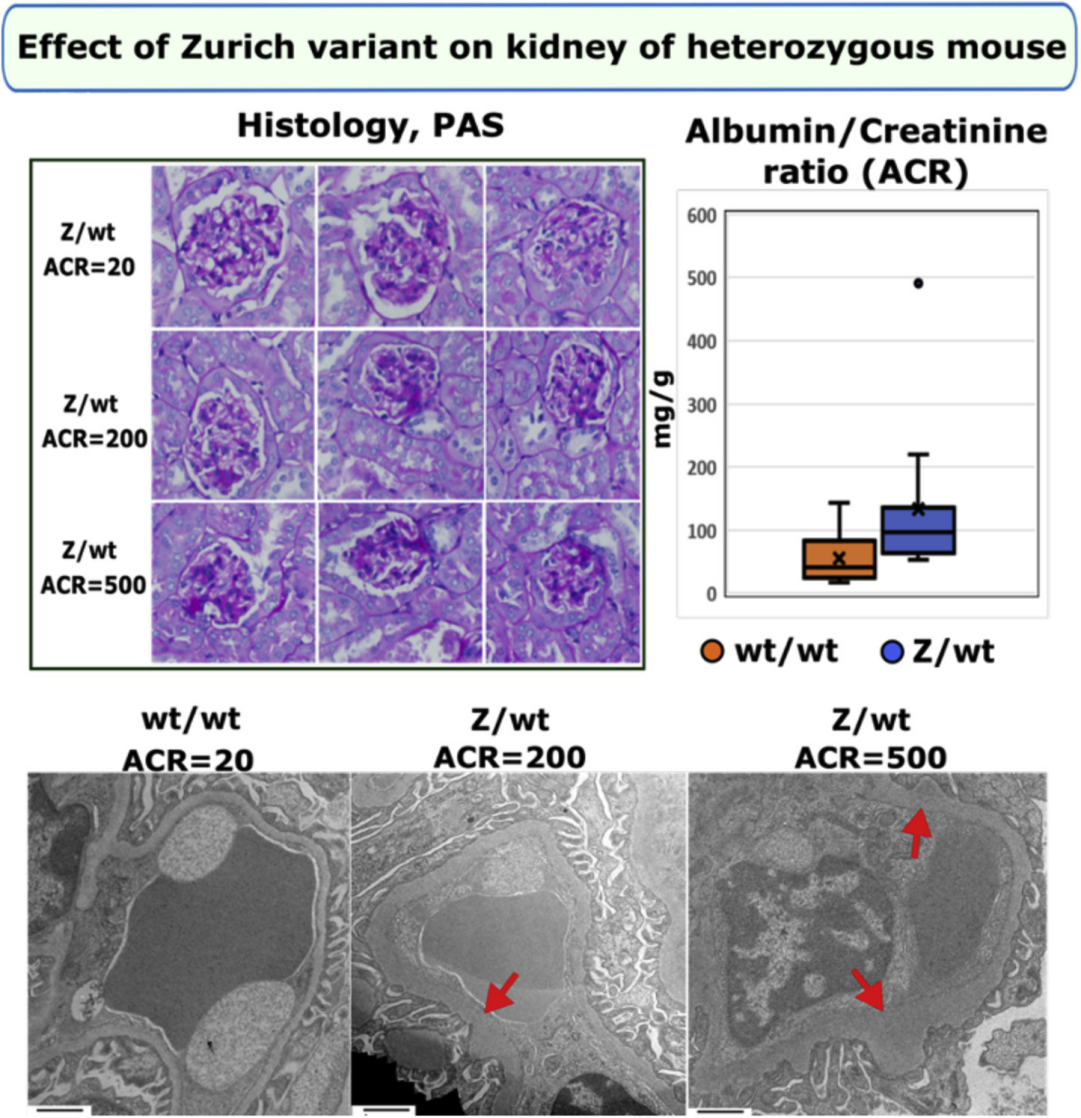
**Zurich variant knock-in heterozygous mouse (Col4a3**^**z/+**^**mouse) shows pathologic phenotype in kidneys at 1 year of age.** Histological analysis of periodic acid–Schiff (PAS)-stained kidney sections (×400) revealed varying degree of glomerular sclerosis (*top left*). Representative transmission electron microscopy (TEM) images of the glomerular capillary loops from two different Col4a3^z/+^ mice and a wild-type control mouse are shown at the *bottom*. The glomerular basement membrane (GBM) was irregularly thickened in Col4a3^z/+^ mice (*red arrows*; TEM images scale bars are 1 μm). One-year-old Col4a3^z/+^ mice (n = 12) exhibited mild albuminuria compared with the age-matched WT mice (n = 9). Albumin-to-creatinine ratios are represented in graph (*top right*). Both experimental groups included male and female mice. The *p* value was 0.04.

Histological analysis of kidney sections revealed glomerulosclerosis with rare crescents in homozygous Col4a3^z/z^ mice (Fig. 4). The GBM was irregular in thickness, lamellated with occasional splitting and with podocyte foot processes effacement while morphometry revealed substantial and widely variable GBM thickening (Fig. 4). The majority of Zurich mice developed moderate albuminuria from 9 to 23 weeks (Fig. 4 and Fig. S7), while two mice displayed a rapid tenfold increase in urine albumin-to-creatinine ratios at 14 to 16 weeks (Fig. S8), demonstrating variability in the appendage effects. No circulating α3NC1-specific antibodies were detected (Fig. S9). Aged heterozygous Col4a3^+/z^ mice also displayed irregular thickened GBM and mild but significant albuminuria (Fig. 5). This heterozygous phenotype is distinct from the heterozygous knock-out model, which lacks the phenotype (47, 48). The difference highlights the toxicity of the Z-appendage in the function of the collagen IV^α345^ scaffold.

Collectively, these findings revealed that the Z-appendage rendered the α345 hexamer defective causing a wide range of ultrastructural abnormalities in the GBM, proteinuria, and glomerulosclerosis. Animals with high level of albuminuria displayed GBM changes characteristic for AS while moderately affected mice had GBM thickening. The mild phenotype observed in the heterozygous mice recapitulates the dominant trait in humans and suggests that defective α345 hexamer “signals” the pathological changes. In contrast to knock-out mouse models that eliminate the collagen IV^α345^ scaffold from basement membranes, the Zurich mouse provides trackability to discover functions of the α345 scaffold at a specific site and to discover pathogenic mechanisms. The absence of α3NC1 autoantibodies indicates that the appendage is not causal, but a risk factor in GP disease. The Z-appendage pathogenicity, in both homozygous and heterozygous mice, pinpointed the α345 hexamer as a critical structure, enabling the GBM to function as a permselective filter.

## Discussion

In the present study of two rare diseases, familial GP and AS, a genetic collagen IV^α345^ variant encoding a short 8-residue appendage, common to both diseases, was found to serve as a beacon into the inner workings of the GBM. The appendage is pathogenic in Zurich mice, causing ultrastructural abnormalities in the GBM, albuminuria, and glomerulosclerosis. These features phenocopied the wide spectrum of glomerular phenotypes in human AS, which supports the recent nomenclature of *COL4A3*, *COL4A4*, and *COL4A5* variants as AS (37). Moreover, the prevalence of the Z-variant is estimated to be approximately one million people worldwide, placing them at risk of progressing to renal failure and developingGP (Fig. 2*A*).

**Figure 6:**
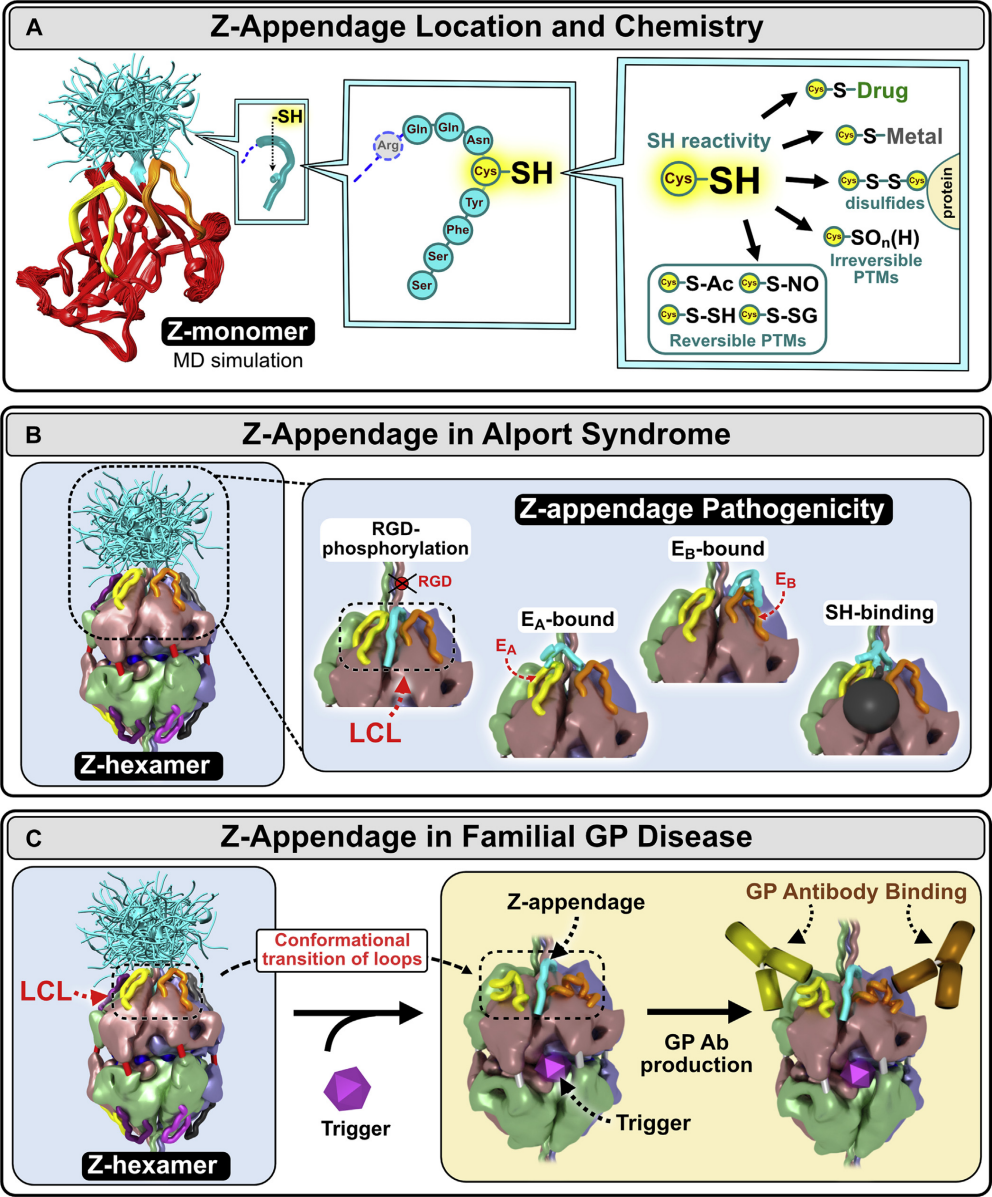
**Z-appendage can participate in multiple chemical interactions and thus can contribute to AS and GP pathogenesis.***A*, the Z-appendage is an 8-residue extension of the α3 chain replacing the C-terminal histidine residue of the native structure. It is located at the apex of α3NC1 monomer, in juxtaposition with E_A_ and E_B_ hypoepitope loops (*left*). The appendage is flexible and can assume multiple conformations as predicted by molecular dynamics (MD) simulations (*left*). Z-appendage features a cysteine residue with free sulfhydryl group (*middle*). This group can participate in multiple reactions resulting in posttranslational modifications (PTMs), complexes with metal ions or small molecule drugs, and disulfide bonds with proteins (*right*) (49, 50). *B*, the appendage has been predicted to assume multiple configurations (*left*); not shown is the Z-appendage of the opposing α345 trimer. Because of its relatively short length, the expected impact of Z-appendage is on the structure and/or function of a specific area within the α345 hexamer including the E_A_ and E_B_ hypoepitopes and the crevice between them called the loop-crevice-loop (LCL) site. In the pathogenesis of Alport syndrome, Z-appendage can block the RGD integrin binding site (60) or phosphorylation site (61) located in the adjacent triple helical domain, interfere with the E_A_ and E_B_ hypoepitope loops, and attract toxic small molecules and metals to the crevice (*inset*). *C*, in Goodpasture’s (GP) disease, Z-appendage can sensitized α345 hexamer, a GP autoantigen, to different second-hit triggers of GP autoantibody production such as environmental toxins or endogenous pathogenic factors, *e.g.*, inflammatory response to bacterial infections, glycoxidative stress in diabetes, etc.

**Figure 7:**
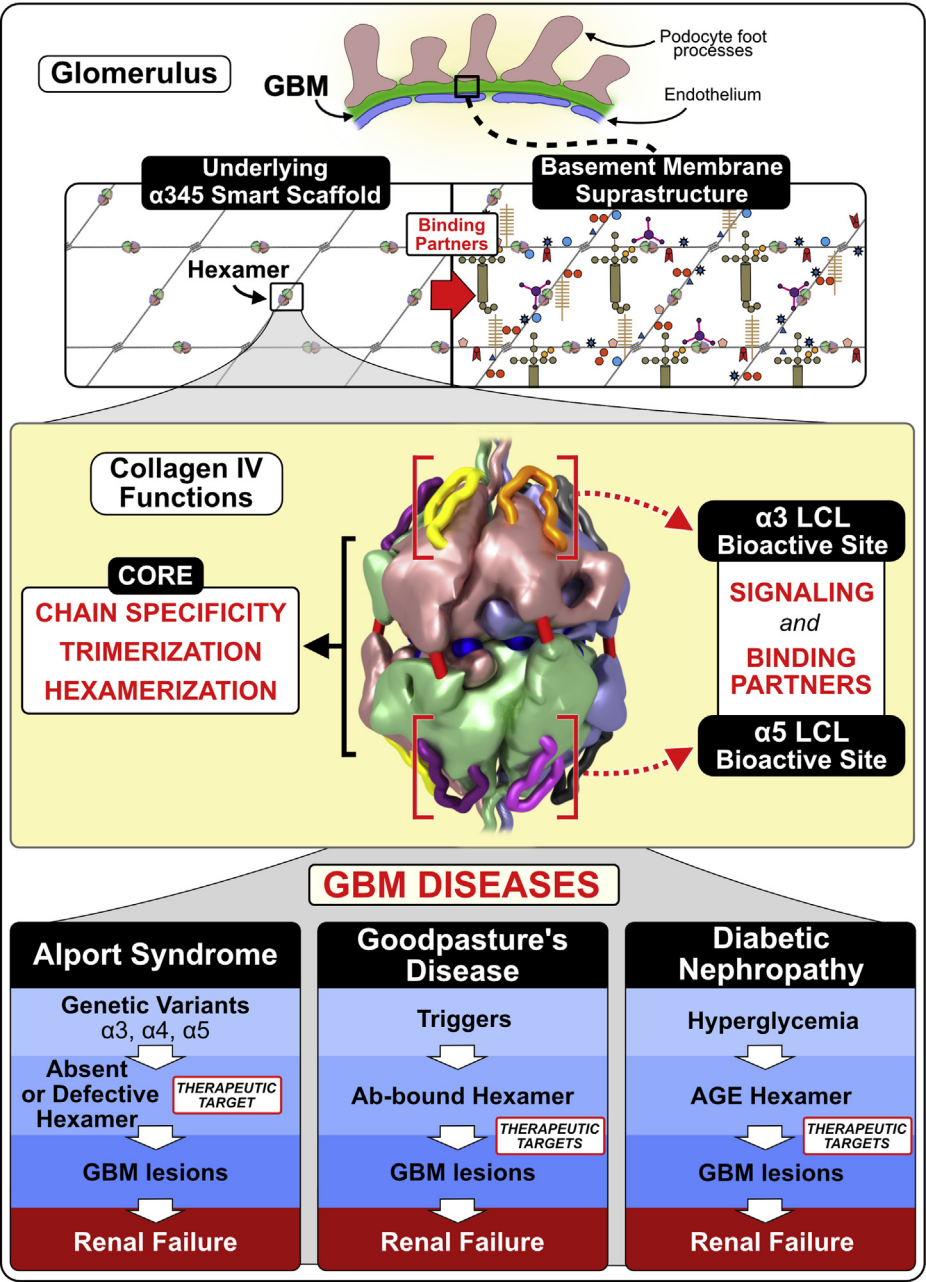
**The α345 hexamer is a focal point in GBM function and dysfunction with LCL sites as potential therapeutic targets in the treatment of GBM kidney diseases.** The collagen IV^α345^ scaffold is a major structure underlying glomerular (GBM) basement membrane. In the GBM, it is deposited by podocytes (62) in the form of protomers that self-assemble in the presence of extracellular levels of chloride ions (63). The scaffold has been defined as a “smart” scaffold (64) due to the presence of multiple binding sites and surfaces that allow participation in quaternary and quinary interactions (65) within the crowded macromolecular environment of the insoluble basement membrane. The α345 hexamer is a key connection module within the collagen IV^α345^ scaffold. The noncollagenous (NC1) domains of individual α-chains forming the hexamer encode its specific composition and assembly *via* intracellular trimerization followed by extracellular hexamerization. Quaternary and quinary interactions involving the hexamer surface may include binding partners within the basement membrane and cell surface receptors inducing signaling. There are functional loop-crevice-loop (LCL) bioactive sites at the apices of the hexameric structure (indicated by *red square brackets*) where pathogenic mechanisms for Alport syndrome, Goodpasture’s disease, and potentially diabetic nephropathy converge. This convergence of pathogenic pathways indicates that the LCL site harbors bioactive functions, including signaling and organizing macromolecular complexes, which underlie the GBM biology. The LCL sites are targets for genetic variants and toxic triggers causing basement membrane abnormalities and leading to renal, pulmonary, otic, and eye disorders. Triggers are envisioned as both environmental and endogenous, *e.g.*, hyperglycemia in diabetes. The LCL sites and downstream pathways are potential targets for rational design of protein replacement and small-molecule therapies. For additional details, see Boudko *et al.* (49), Fig. 6.

The crystal structure and assembly mechanisms of the α345 hexamer, as presented in Boudko *et al.* (49) and Pedchenko *et al.* (50), provided a framework to interpret how the Z-appendage, a representative AS variant, played a role in AS and a possible structural risk factor for GP (49, 50). Z-appendage features a cysteine residue with free sulfhydryl group. This group can participate in multiple reactions resulting in posttranslational modifications (PTMs), complexes with metal ions or small molecule drugs, and disulfide bonds with proteins (Fig. 6*A* and Boudko *et al.* 49) (51, 52). The appendage is juxtaposed with the GP hypoepitopes forming common “hotspots” of dysfunction, called the LCL site (49). This convergence of pathogenic pathways indicates that the LCL site is bioactive, having functions that include signaling and organizing macromolecular complexes (49, 50). Since the LCL bioactive site is conformationally plastic (50), its functions can be perturbed by the Z-appendage and other genetic variants that occur in the hexamer in AS (Fig. 6*B*), endogenous and exogenous triggers in GP (Fig. 6*C*), and hyperglycemia in DN (Fig. 7 and Boudko *et al.* 49). Therefore, we conclude that the α345 hexamer is a focal point with bioactivity, enabling GBM morphology and its function as a permselective filter, which can be altered in GBM diseases.

Collectively, these findings demonstrate that the α345 hexamer is a promising target for therapeutic intervention. Attractive therapeutic strategies for AS include protein replacement and pharmacological chaperones, because the GBM is directly accessible to protein delivery *via* the bloodstream. Recent advances in delivery of full-length recombinant laminin molecules to the GBM (53) set up a possibility that a full-length or mini-α345 protomer can be delivered therapeutically to the glomerulus, where it can oligomerize forming the collagen IV^α345^ scaffold in the GBM (50). Moreover, because a significant number of hypomorph variants occurs within the hexamer (49), the multiple pores, crevices, and cavities of the α345 hexamer can be potential targets for pharmacological chaperone interventions to correct misfolding or stabilize the protein and reduce degradation. In addition, the knock-in mouse is a promising model for the development of new drugs and gene editing therapies for AS.

## Experimental procedures

### Human subjects

All human studies were done in accordance with the Helsinki Principles. Informed consent was obtained from all patients included in the studies, and the Institutional Review Board of the Vanderbilt University Medical Center and the Cantonal Ethics Committee of Zurich approved this study.

### Genomic DNA extraction and library preparation

Genomic DNA from FFPE samples was extracted with either Covaris of QIAGEN FFPE DNA extraction kits. DNA from blood cells was extracted with Promega’s Wizard Genomic DNA kit (cat# A1120).

Whole-genome libraries were prepared from genomic DNA with the NEBNext Ultra II DNA Library Prep Kit for Illumina per manufacturer’s instructions. The DNA was fragmented on the Covaris LE220 targeting an average insert size of 400 to 500 bp. The blunt ends of each sample were end repaired, adenylated for adaptor ligation, ligated to standard Illumina adapters, and PCR amplified.

Whole-exome libraries were generated from genomic DNA extracted from FFPE kidney biopsies using the Exome captured with IDT xGen Exome Research Panel library (IDT).

### Whole-exome and whole-genome data analysis

Both whole exome sequence and whole genome sequence were subjected to DNA sequencing on the NovaSeq Illumina platform, and genotype calls were made at targeted bases as described below (Zurich section). All targeted bases of COL4A3 exon 52 in WES experiments show ≥20 independent reads in all samples and were subject for *de novo* mutation detection using human reference genome hg19. Average sequencing depth for all bases in targeted region (COL4A3 exon 52) in WGS experiments was at least ×50.

### Library sequencing

Captured libraries were sequenced using the Illumina NovaSeq 6000 system (Illumina) with paired end reads of 151 bp according to the manufacturer’s protocols. Raw reads in FASTQ format from WES or WGS were aligned to the hg19 reference genome using the Burrows–Wheeler Aligner (BWA; http://bio-bwa.sourceforge.net/). Duplicates were removed with Picard (http://picard.Sourceforget.net). WES and WGS data were analyzed using GATK INDEL calling algorithm (GATK (http://www.broadinstitute.org/gatk/) following the guidelines provided in the user manuals.

### SNPs filtration and annotation

INDELs and the variants were filtered using GATK and annotated using the ANNOVAR program (http://www.openbioinformatics.org/annovar/).

### Production of reagents for immunoassays of GP patient autoantibodies

Recombinant human α1-α6NC1 monomers and α3/α1 monomeric chimeras bearing E_A_ and E_B_ regions of α3NC1 were purified from the culture medium of stably transfected HEK 293 cells using anti-FLAG agarose (31, 54). For the construction of mutated α3NC1 or α1NC1 domains containing an 8-residue extension on carboxy termini, we used PCR mutagenesis with corresponding pRcX expression vectors as template (55). Introduction of the target mutations was verified by automated sequencing. Native collagen IV NC1 hexamers were isolated from human and bovine GBM after collagenase digestion (55). In some experiments, the GBM NC1 hexamers were denatured by treatment with 6 mol/l guanidine-HCl for 30 min at 60^o^ C prior to coating on ELISA plates.

### NC1 domain isolation from mouse tissues and western blotting

The kidneys or lungs from wild-type control and homozygous Zurich mutant mice were homogenized using metal bug beads homogenizer system in TBS buffer containing 0.1% Tween 20 (TBST). Beads were centrifuged and precipitated material was washed with TBST four times. At the final step precipitate was dissolved in 250 μl of collagenase digestion buffer (55) containing 100 μg/ml of collagenase and incubated overnight at 37 °C. Soluble portion was loaded and separated by SDS-PAGE using 4 to 20% gradient gel, transferred to nitrocellulose membranes for probing with H31, H43, and Mab5 antibodies.

### Mouse tissue immunofluorescence and light microscopy analysis

Kidneys were cut and dropped into near-freezing pentane (Fisher Scientific). Lungs were first inflated with 1:1 solution of optimal cutting temperature compound (Tissue-Tek) and PBS. For cryosectioning, tissue was embedded into the mold and 4 μm sections were cut with cryostat and placed on slides. Slides were pretreated with 6 M urea in 0.1 M glycine-HCl buffer, pH 3.0, for 10 min (24), followed by several washes with PBS and PBS/0.2% Tween. Slides were preincubated with 10% normal goat serum (Invitrogen) for 1 h at RT to block nonspecific binding of antibodies. The following primary antibodies were used for antigen detections: rat anti-collagen IV α3 NC1 (1:250 dilution, H31), rat anti-collagen IV α4 NC1 (1:250 dilution, H43), and mouse anti-collagen IV α5 NC1 (1:250, Mab5). The H31 and H43 antibodies were from Y. Sado (Shigei Medical Research Institute). The secondary antibodies used for immunofluorescence detection were Alexa555 goat anti-rat (1:1000 dilution; Abcam) and Alexa568 goat anti-mouse (1:1000 dilution; Abcam). All antibodies were diluted in PBS/0.1% Tween and 5% normal goat serum. Slides were incubated with primary antibodies overnight at 4 °C in a humidified chamber and then washed three times in PBS/0.2% Tween before incubating with secondary antibodies for 1 h at RT. Negative control slide was processed similarly to experimental slide, but without primary antibodies.

For light microscopic examination, 5-μm paraffin sections of the lung tissue (n = 4 for each genotype) were stained with Periodic acid–Schiff and hematoxylin and eosin. Slides were reviewed by a pathologist in a blinded fashion and the morphometric data were acquired and subjectively analyzed. Morphometric analysis was performed as described previously (56).

### Mouse urine samples collection and ACR measurements

Urine samples were collected according to animal protocol (M1900063-00) approved by VUMC IACUC. Albumin concentrations were measured with Albuwell M kit (Exocell, Inc) and creatinine concentrations were determined with Creatinine Companion kit (Exocell, Inc) according to the protocols provided by the manufacturer. Electrophoresis SDS-PAGE analysis of 3 μl urine was also performed on most samples.

### Transmission electron microscopy of mouse tissues

Freshly extracted kidneys were cut in small pieces (2 × 2 mm) and fixed in 2.5% glutaraldehyde buffered in 0.1 M sodium cacodylate buffer, pH 7.5 overnight. Lungs were inflated with 2 ml of 2% PFA plus 2% glutaraldehyde and left immersed in the same fixative solution overnight. Small pieces were cut next day. Tissues were postfixed in 1% osmium tetroxide, followed by dehydration through a grade series of ethanol to 100%. Samples were further dehydrated in propylene oxide and infiltrated and embedded in Spurr’s epoxy. 70-nm ultrathin sections were collected on 300 mesh copper grids and stained with 2% uranyl acetate followed by Reynold’s lead citrate. Stained sections were examined using a T-12 electron microscope (Philips/FEI) operated at 100 kV and photographed using a 2K camera (AMT).

### Expression and purification of α3, α4, and α5 NC1 monomers

Recombinant human NC1 domains were amplified by PCR from human kidney cDNA library, cloned in derivative of pRc-CMV mammalian expression vector that includes BM-40 signal peptide and N-terminal FLAG tag, and transfected into HEK-293 cells using HEPES-calcium phosphate (ProFection, Promega). Stable clones were selected using neomycin (0.4 mg/ml) and clones with highest levels of NC1 expression after testing by western blotting were expanded into T225 culture flasks. Conditioned medium was collected from confluent cultures two times a week and recombinant proteins were purified by passing through anti-FLAG M2-agarose (Sigma) columns with subsequent elution with FLAG peptide (100 μg/ml, Sigma) and concentration on ultrafiltration concentrators (Amicon 10MWCO, Millipore) to 2 to 4 mg/ml. Proteins were further purified by SEC on Superdex 200 column in TBS buffer (55).

## Data availability

All data described in this article is available in the main text or supporting information.

## Supporting information

This article contains supporting information.

## Conflict of interest

The authors declare that there is no conflict of interest.

## Supporting information

Supplementary Table S2

Supplementary Sections 1–6, Figures S1–S9 and Tables S1, S3
