# Supplementary material for: Collagen IVα345 dysfunction in glomerular basement membrane diseases. I. Discovery of a COL4A3 variant in familial Goodpasture’s and Alport diseases
Source: J Biol Chem. 2021 Mar 26;296:100590. doi: 10.1016/j.jbc.2021.100590 (PMC8100070; doi:10.1016/j.jbc.2021.100590)
Supplement: Supplementary Table S2 [file mmc1.pdf]

Supplemental Table S2. The list of Goodpasture's disease patients (familial and sporadic).

GP patients

China cohort (171): Anti-GBM disease was defined as positive anti-GBM antibodies in circulation and/or linear immunoglobulin G deposition along the GBM on kidney biopsy. [Cui, Z. et al. Am J Kidney Dis 2011 57 4 575-82 PMID=21168945]
USA cohort (37): Patients were diagnosed with GP disease if they were positive for 1) glomerular crescents and 2) linear anti-GBM staining
UK cohort (29) for criteria please see table below.
Sweden cohort (15): 1) circulating anti-GBM antibodies, 2) clinical history of rapidly progressive glomerulonephritis [Persson, U., Hertz, J. M., Carlsson, M., Hellmark, T., Juncker, I., Wieslander, J. and Segelmark, M. Nephrol Dial Transplant 2004 19 8 2030-5 PMID=15199166]
Swiss cohort (11): Persson, U., Hertz, J. M., Carlsson, M., Hellmark, T., Juncker, I., Wieslander, J. and Segelmark, M. Nephrol Dial Transplant 2004 19 8 2030-5 PMID=15199166

Patient	Clinical diagnosis	Geographical location	Cohort	Gender	Age (DOB) at prese ntatio	Race	Genotyping WGS/WES/Sanger	Genotype	Kidney abnormalities	Lung abnormalities	Tissue bound anti-GBM	Circulating anti-GBM (U/ml)	ANCA	Family history of kidney disease
Zurich-01 (Index)	GP	Switzerland	Dr. Seeger	M	23	W	WGS/Sanger	.5010_*del (p.His1670_*167delinsGln*9)	not biopsied	diffuse alveolar hemorrhage	NT	151	NO	YES
Zurich-02	GP	Switzerland	Dr. Seeger	F	45	W	WGS/Sanger	.5010_*del (p.His1670_*167delinsGln*9)	Diffuse extracapillary proliferation	diffuse alveolar hemorrhage	linear staining	72	NO	YES
31 e	GP	Switzerland	Dr. Seeger	M	22	A	Sanger	WT	Focal extracapillary proliferation	diffuse alveolar hemorrhage	linear staining	541	no	no
32 f	GP	Switzerland	Dr. Seeger	M	22	W	Sanger	WT	Diffuse extracapillary proliferation	diffuse alveolar hemorrhage	linear staining	1099	no	no
33 g	GP	Switzerland	Dr. Seeger	F	38	W	Sanger	WT	Diffuse extracapillary proliferation	diffuse alveolar hemorrhage	linear staining	31	no	no
34 h	GP	Switzerland	Dr. Seeger	M	37	W	Sanger	WT	Diffuse extracapillary proliferation	none	linear staining	736	no	no
56d	GP	Switzerland	Dr. Seeger	M	22	W	Sanger	WT	Diffuse extracapillary proliferation	none	and granular st	12	no	no
36j	GP	Switzerland	Dr. Seeger	F	60	W	Sanger	WT	Diffuse extracapillary proliferation	none	linear staining	360	no	no
37k	GP	Switzerland	Dr. Seeger	F	22	W	Sanger	WT	report not available	diffuse alveolar hemorrhage	NA	positive	no	no
38l	GP+ANCA	Switzerland	Dr. Seeger	F	73	As	Sanger	WT	Diffuse extracapillary proliferation	none	linear staining	63	yes	no
54b	GP	Switzerland	Dr. Seeger	F	58	W	Sanger	WT	Diffuse extracapillary proliferation	none	linear staining	positive	no	no
zh50	GP	Switzerland	Dr. Seeger	M	19	W	Sanger	WT	Diffuse extracapillary proliferation	none	linear staining	3200	no	no
dsp	GP+ANCA	Switzerland	Dr. Seeger	M	48	W	Sanger	WT	Diffuse extracapillary proliferation	diffuse alveolar hemorrhage	ar/granular stai	95	yes	no
LL 6-20-18	GP	USA	Dr Hudson (VP)				WGS	WT	glomerular crescent		linear staining			
GP-AFI	GP	USA	Dr Hudson (VP)				WGS	WT	glomerular crescent		linear staining			
Mayo clinic	GP	USA	Dr. Fervenza	M			WGS	WT	?					YES
K81-129	GP	USA	Dr. Fogo	F			WES	WT	glomerular crescent		linear staining			
K91-31	GP	USA	Dr. Fogo	M			WES	WT	glomerular crescent		linear staining			
K91-54	GP	USA	Dr. Fogo	F	54		WES	WT	glomerular crescent		linear staining			
K02-170	GP	USA	Dr. Fogo	M	26	W	WES	WT	glomerular crescent		linear staining			
K06-75	GP	USA	Dr. Fogo	F	47		WES	WT	glomerular crescent		linear staining			
K07-818	GP	USA	Dr. Fogo	M	34		WES	WT	glomerular crescent		linear staining			
14P00218	GP	USA	Dr. Fogo				WES	WT	glomerular crescent		linear staining			
K08-88	GP	USA	Dr. Fogo	M	83		WES	WT	glomerular crescent		linear staining			
K08-227	GP	USA	Dr. Fogo	M	20	W	WES	WT	glomerular crescent		linear staining			
K09-620	GP	USA	Dr. Fogo	M	23		WES	WT	glomerular crescent		linear staining			
K09-707	GP	USA	Dr. Fogo	M	58	W	WES	WT	glomerular crescent		linear staining			
K09-1057	GP	USA	Dr. Fogo	M	79	W	WES	WT	glomerular crescent		linear staining			
K10-165	GP	USA	Dr. Fogo	F	77		WES	WT	glomerular crescent		linear staining			
K10-459	GP	USA	Dr. Fogo	M	34	W	WES	WT	glomerular crescent		linear staining			
K11-141	GP	USA	Dr. Fogo	F	9	As	WES	WT	glomerular crescent		linear staining			
K11-1039	GP	USA	Dr. Fogo	F	8	W	WES	WT	glomerular crescent		linear staining			
K13-631	GP	USA	Dr. Fogo	F	49	W	WES	WT	glomerular crescent		linear staining			
K13-978	GP	USA	Dr. Fogo	M	24		WES	WT	glomerular crescent		linear staining			
K13-1208	GP	USA	Dr. Fogo	F	65	W	WES	WT	glomerular crescent		linear staining			
K14-364	GP	USA	Dr. Fogo	M	70	W	WES	WT	glomerular crescent		linear staining			
K15-923	GP	USA	Dr. Fogo	M	53	W	WES	WT	glomerular crescent		linear staining			
K15-1040	GP	USA	Dr. Fogo	M	68		WES	WT	glomerular crescent		linear staining			
K15-1109	GP	USA	Dr. Fogo	F	61	W	WES	WT	glomerular crescent		linear staining			
K16-164	GP	USA	Dr. Fogo	M	64		WES	WT	glomerular crescent		linear staining			
K16-170	GP	USA	Dr. Fogo	M	35		WES	WT	glomerular crescent		linear staining			
K16-461	GP	USA	Dr. Fogo	M	66		WES	WT	glomerular crescent		linear staining			
K17-349	GP	USA	Dr. Fogo	F	71	AA	WES	WT	glomerular crescent		linear staining			
K17-719	GP	USA	Dr. Fogo	M	57	W	WES	WT	glomerular crescent		linear staining			
K17-844	GP	USA	Dr. Fogo	F	37		WES	WT	glomerular crescent		linear staining			
K17-1003	GP	USA	Dr. Fogo	M	38		WES	WT	glomerular crescent		linear staining			
K18-271	GP	USA	Dr. Fogo	M	55		WES	WT	glomerular crescent		linear staining			
K18-378	GP	USA	Dr. Fogo	F	48	W	WES	WT	glomerular crescent		linear staining			
T01-1787	GP	UK	Dr. Mc Adoo	F	42	uth Asi	Sanger	WT	no biopsy	Alveolar Haemorrhage	no biopsy	positive	egative	
T02-947	GP	UK	Dr. Mc Adoo	F	66	White	Sanger	WT	Crescentic GN	None	linear IgG	positive	MPO	
T04-139	GP	UK	Dr. Mc Adoo	M	68	White	Sanger	WT	Crescentic GN	None	linear IgG	positive	neg	
T05-105	GP	UK	Dr. Mc Adoo	F	63	White	Sanger	WT	no biopsy	None	no biopsy	positive	neg	
T06-2726	GP	UK	Dr. Mc Adoo	F	74	White	Sanger	WT	Crescentic GN	None	linear IgG	92	neg	
T07-1406	GP	UK	Dr. Mc Adoo	M	25	White	Sanger	WT	Crescentic GN	None	linear IgG	seronegative	neg	
T07-1531	GP	UK	Dr. Mc Adoo	F	71	uth Asi	Sanger	WT	Crescentic GN	None	linear IgG	185	MPO	
T08-164	GP	UK	Dr. Mc Adoo	F	52	uth Asi	Sanger	WT	no biopsy	None	no biopsy	positive	neg	
T08-1352	GP	UK	Dr. Mc Adoo	M	58	ansplar	Sanger	WT	Crescentic GN	Alveolar Haemorrhage	linear IgG	30	neg	
T10-2956 = 10--24	GP	UK	Dr. Mc Adoo	M	46	-Caribb	Sanger	WT	Crescentic GN	Alveolar Haemorrhage	linear IgG	59	PR3	
T11-2171	GP	UK	Dr. Mc Adoo	F	59	uth Asi	Sanger	WT	Crescentic GN	None	linear IgG	seronegative	neg	
1 11--2988	GP	UK	Dr. Mc Adoo	F	47	White	Sanger	WT	no biopsy	None	no biopsy	positive	neg	
12-504	GP	UK	Dr. Mc Adoo	F	36	White	Sanger	WT	Normal LM	Alveolar Haemorrhage	linear IgG	positive	neg	
13-044	GP	UK	Dr. Mc Adoo	F	62	White	Sanger	WT	no biopsy	None	no biopsy	294	neg	
13-716	GP	UK	Dr. Mc Adoo	F	48	White	Sanger	WT	no biopsy	None	no biopsy	165	MPO	
14-4348	GP	UK	Dr. Mc Adoo	M	70	White	Sanger	WT	Crescentic GN	Alveolar Haemorrhage	linear IgG	196	neg	
16--1154	GP	UK	Dr. Mc Adoo	F	63	uth Asi	Sanger	WT	Crescentic GN	None	linear IgG	serongative	MPO	
16--1166	GP	UK	Dr. Mc Adoo	F	75	uth Asi	Sanger	WT	Crescentic GN	Alveolar Haemorrhage	pauci-immune	130	MPO	
16-1195	GP	UK	Dr. Mc Adoo	F	56	uth Asi	Sanger	WT	Crescentic GN	None	linear IgG	196	MPO	
17-5075	GP	UK	Dr. Mc Adoo	F	53	uth Asi	Sanger	WT	Crescentic GN	None	linear IgG	positive	MPO	
11-335	GP	UK	Dr. Mc Adoo	F	70	White	Sanger	WT	no biopsy	None	no biopsy	positive	neg	
17-3777 = 15--357	GP	UK	Dr. Mc Adoo	F	67	uth Asi	Sanger	WT	Crescentic GN	Alveolar Haemorrhage	linear IgG	295	PR3	
19--01	GP	UK	Dr. Mc Adoo	F	37	White	Sanger	WT	Crescentic GN	Alveolar Haemorrhage	linear IgG	16	PR3	
19--02	GP	UK	Dr. Mc Adoo	M	47	White	Sanger	WT	Crescentic GN	None	linear IgG	346	neg	
19--03	GP	UK	Dr. Mc Adoo	F	29	White	Sanger	WT	Crescentic GN	None	linear IgG	393	neg	
19--04	GP	UK	Dr. Mc Adoo	M	49	White	Sanger	WT	Crescentic GN	None	linear IgG	97	neg	
19--05	GP	UK	Dr. Mc Adoo	M	44	White	Sanger	WT	Crescentic GN	None	gloms in IF sam	77	neg	
19--06	GP	UK	Dr. Mc Adoo	F	59	White	Sanger	WT	Crescentic GN	None	linear IgG	27	PR3	
19--07	GP	UK	Dr. Mc Adoo	F	28	White	Sanger	WT	Crescentic GN	None	linear IgG	220	neg	
09--2072	GP	UK	Dr. Mc Adoo	F	48	White	Sanger	WT	Crescentic GN	Alveolar Haemorrhage	linear IgG	201	neg	
EX053-1	GP	China	Dr. Cui				Sanger	WT						
EX053-2	GP	China	Dr. Cui				Sanger	WT						
EX053-3	GP	China	Dr. Cui				Sanger	WT						
EX053-5	GP	China	Dr. Cui				Sanger	WT						
EX053-6	GP	China	Dr. Cui				Sanger	WT						
EX053-7	GP	China	Dr. Cui				Sanger	WT						
EX053-8	GP	China	Dr. Cui				Sanger	WT						
EX053-9	GP	China	Dr. Cui				Sanger	WT						
EX053-10	GP	China	Dr. Cui				Sanger	WT						
EX053-11	GP	China	Dr. Cui				Sanger	WT						
EX053-12	GP	China	Dr. Cui				Sanger	WT						
EX053-14	GP	China	Dr. Cui				Sanger	WT						
EX053-15	GP	China	Dr. Cui				Sanger	WT						
EX053-16	GP	China	Dr. Cui				Sanger	WT						
EX053-17	GP	China	Dr. Cui				Sanger	WT						
EX053-18	GP	China	Dr. Cui				Sanger	WT						
EX053-19	GP	China	Dr. Cui				Sanger	WT						
EX053-20	GP	China	Dr. Cui				Sanger	WT						
EX053-23	GP	China	Dr. Cui				Sanger	WT						
EX053-24	GP	China	Dr. Cui				Sanger	WT						
EX053-25	GP	China	Dr. Cui				Sanger	WT						
EX053-26	GP	China	Dr. Cui				Sanger	WT						
EX053-27	GP	China	Dr. Cui				Sanger	WT						
EX053-28	GP	China	Dr. Cui				Sanger	WT						
EX053-29	GP	China	Dr. Cui				Sanger	WT						
EX053-30	GP	China	Dr. Cui				Sanger	WT						
EX053-32	GP	China	Dr. Cui				Sanger	WT						
EX053-35	GP	China	Dr. Cui				Sanger	WT						
EX053-36	GP	China	Dr. Cui				Sanger	WT						
EX053-37	GP	China	Dr. Cui				Sanger	WT						
EX053-38	GP	China	Dr. Cui				Sanger	WT						
EX053-39	GP	China	Dr. Cui				Sanger	WT						
EX053-41	GP	China	Dr. Cui				Sanger	WT						
EX053-42	GP	China	Dr. Cui				Sanger	WT						
EX053-43	GP	China	Dr. Cui				Sanger	WT						
EX053-44	GP	China	Dr. Cui				Sanger	WT						
EX053-45	GP	China	Dr. Cui				Sanger	WT						
EX053-46	GP	China	Dr. Cui				Sanger	WT						
EX053-47	GP	China	Dr. Cui				Sanger	WT						
EX053-48	GP	China	Dr. Cui				Sanger	WT						
EX053-49	GP	China	Dr. Cui				Sanger	WT						
EX053-50	GP	China	Dr. Cui				Sanger	WT						
EX053-51	GP	China	Dr. Cui				Sanger	WT						
EX053-52	GP	China	Dr. Cui				Sanger	WT						
EX053-53	GP	China	Dr. Cui				Sanger	WT						
EX053-54	GP	China	Dr. Cui				Sanger	WT						
EX053-55	GP	China	Dr. Cui				Sanger	WT						
EX053-57	GP	China	Dr. Cui				Sanger	WT						
EX053-58	GP	China	Dr. Cui				Sanger	WT						
EX053-59	GP	China	Dr. Cui				Sanger	WT						
EX053-60	GP	China	Dr. Cui				Sanger	WT						
EX053-62	GP	China	Dr. Cui				Sanger	WT						
EX053-64	GP	China	Dr. Cui				Sanger	WT						
EX053-66	GP	China	Dr. Cui				Sanger	WT						
EX053-67	GP	China	Dr. Cui				Sanger	WT						
EX053-68	GP	China	Dr. Cui				Sanger	WT						
EX053-69	GP	China	Dr. Cui				Sanger	WT						
EX053-70	GP	China	Dr. Cui				Sanger	WT						
EX053-71	GP	China	Dr. Cui				Sanger	WT						
EX053-72	GP	China	Dr. Cui				Sanger	WT						
EX053-73	GP	China	Dr. Cui				Sanger	WT						
EX053-74	GP	China	Dr. Cui				Sanger	WT						
EX053-75	GP	China	Dr. Cui				Sanger	WT						
EX053-76	GP	China	Dr. Cui				Sanger	WT						
EX053-77	GP	China	Dr. Cui				Sanger	WT						
EX053-79	GP	China	Dr. Cui				Sanger	WT						
EX053-81	GP	China	Dr. Cui				Sanger	WT						
EX053-85	GP	China	Dr. Cui				Sanger	WT						
EX053-87	GP	China	Dr. Cui				Sanger	WT						
EX053-88	GP	China	Dr. Cui				Sanger	WT						
EX053-89	GP	China	Dr. Cui				Sanger	WT						
EX053-90	GP	China	Dr. Cui				Sanger	WT						
EX053-91	GP	China	Dr. Cui				Sanger	WT						
EX053-92	GP	China	Dr. Cui				Sanger	WT						
EX053-93	GP	China	Dr. Cui				Sanger	WT						
EX053-94	GP	China	Dr. Cui				Sanger	WT						
EX053-95	GP	China	Dr. Cui				Sanger	WT						
EX053-96	GP	China	Dr. Cui				Sanger	WT						
EX053-97	GP	China	Dr. Cui				Sanger	WT						

EX053-98	GP	China	Dr. Cui				Sanger	WT						
EX053-99	GP	China	Dr. Cui				Sanger	WT						
EX053-100	GP	China	Dr. Cui				Sanger	WT						
EX053-102	GP	China	Dr. Cui				Sanger	WT						
EX053-103	GP	China	Dr. Cui				Sanger	WT						
EX053-104	GP	China	Dr. Cui				Sanger	WT						
EX053-107	GP	China	Dr. Cui				Sanger	WT						
EX053-108	GP	China	Dr. Cui				Sanger	WT						
EX053-109	GP	China	Dr. Cui				Sanger	WT						
EX053-110	GP	China	Dr. Cui				Sanger	WT						
EX053-111	GP	China	Dr. Cui				Sanger	WT						
EX053-112	GP	China	Dr. Cui				Sanger	WT						
EX053-113	GP	China	Dr. Cui				Sanger	WT						
EX053-114	GP	China	Dr. Cui				Sanger	WT						
EX053-116	GP	China	Dr. Cui				Sanger	WT						
EX053-117	GP	China	Dr. Cui				Sanger	WT						
EX053-118	GP	China	Dr. Cui				Sanger	WT						
EX053-120	GP	China	Dr. Cui				Sanger	WT						
EX053-121	GP	China	Dr. Cui				Sanger	WT						
EX053-124	GP	China	Dr. Cui				Sanger	WT						
EX053-125	GP	China	Dr. Cui				Sanger	WT						
EX053-129	GP	China	Dr. Cui				Sanger	WT						
EX053-130	GP	China	Dr. Cui				Sanger	WT						
EX053-132	GP	China	Dr. Cui				Sanger	WT						
EX053-133	GP	China	Dr. Cui				Sanger	WT						
EX053-134	GP	China	Dr. Cui				Sanger	WT						
EX053-135	GP	China	Dr. Cui				Sanger	WT						
EX053-136	GP	China	Dr. Cui				Sanger	WT						
EX053-137	GP	China	Dr. Cui				Sanger	WT						
EX053-138	GP	China	Dr. Cui				Sanger	WT						
EX053-139	GP	China	Dr. Cui				Sanger	WT						
EX053-140	GP	China	Dr. Cui				Sanger	WT						
EX053-141	GP	China	Dr. Cui				Sanger	WT						
EX053-143	GP	China	Dr. Cui				Sanger	WT						
EX053-145	GP	China	Dr. Cui				Sanger	WT						
EX053-146	GP	China	Dr. Cui				Sanger	WT						
EX053-147	GP	China	Dr. Cui				Sanger	WT						
EX053-148	GP	China	Dr. Cui				Sanger	WT						
EX053-149	GP	China	Dr. Cui				Sanger	WT						
EX053-150	GP	China	Dr. Cui				Sanger	WT						
EX053-151	GP	China	Dr. Cui				Sanger	WT						
EX053-154	GP	China	Dr. Cui				Sanger	WT						
EX053-155	GP	China	Dr. Cui				Sanger	WT						
EX053-156	GP	China	Dr. Cui				Sanger	WT						
EX053-157	GP	China	Dr. Cui				Sanger	WT						
EX053-158	GP	China	Dr. Cui				Sanger	WT						
EX053-159	GP	China	Dr. Cui				Sanger	WT						
EX053-160	GP	China	Dr. Cui				Sanger	WT						
EX053-161	GP	China	Dr. Cui				Sanger	WT						
EX053-162	GP	China	Dr. Cui				Sanger	WT						
EX053-163	GP	China	Dr. Cui				Sanger	WT						
EX053-164	GP	China	Dr. Cui				Sanger	WT						
EX053-167	GP	China	Dr. Cui				Sanger	WT						
EX053-170	GP	China	Dr. Cui				Sanger	WT						
EX053-171	GP	China	Dr. Cui				Sanger	WT						
EX053-172	GP	China	Dr. Cui				Sanger	WT						
EX053-173	GP	China	Dr. Cui				Sanger	WT						
EX053-174	GP	China	Dr. Cui				Sanger	WT						
EX053-175	GP	China	Dr. Cui				Sanger	WT						
EX053-176	GP	China	Dr. Cui				Sanger	WT						
EX053-177	GP	China	Dr. Cui				Sanger	WT						
EX053-178	GP	China	Dr. Cui				Sanger	WT						
EX053-179	GP	China	Dr. Cui				Sanger	WT						
EX053-180	GP	China	Dr. Cui				Sanger	WT						
EX053-181	GP	China	Dr. Cui				Sanger	WT						
EX053-183	GP	China	Dr. Cui				Sanger	WT						
EX053-184	GP	China	Dr. Cui				Sanger	WT						
EX053-185	GP	China	Dr. Cui				Sanger	WT						
EX053-186	GP	China	Dr. Cui				Sanger	WT						
EX053-187	GP	China	Dr. Cui				Sanger	WT						
EX053-188	GP	China	Dr. Cui				Sanger	WT						
EX053-189	GP	China	Dr. Cui				Sanger	WT						
EX053-190	GP	China	Dr. Cui				Sanger	WT						
EX053-191	GP	China	Dr. Cui				Sanger	WT						
EX053-192	GP	China	Dr. Cui				Sanger	WT						
EX053-194	GP	China	Dr. Cui				Sanger	WT						
EX053-195	GP	China	Dr. Cui				Sanger	WT						
EX053-196	GP	China	Dr. Cui				Sanger	WT						
EX053-197	GP	China	Dr. Cui				Sanger	WT						
EX053-198	GP	China	Dr. Cui				Sanger	WT						
EX053-199	GP	China	Dr. Cui				Sanger	WT						
EX053-200	GP	China	Dr. Cui				Sanger	WT						
EX053-201	GP	China	Dr. Cui				Sanger	WT						
EX053-202	GP	China	Dr. Cui				Sanger	WT						
EX053-203	GP	China	Dr. Cui				Sanger	WT						
EX053-204	GP	China	Dr. Cui				Sanger	WT						
EX053-205	GP	China	Dr. Cui				Sanger	WT						
EX053-206	GP	China	Dr. Cui				Sanger	WT						
EX053-207	GP	China	Dr. Cui				Sanger	WT						
EX053-208	GP	China	Dr. Cui				Sanger	WT						
EX053-210	GP	China	Dr. Cui				Sanger	WT						
EX053-211	GP	China	Dr. Cui				Sanger	WT						
