# Supplementary material for: Collagen IVα345 dysfunction in glomerular basement membrane diseases. I. Discovery of a COL4A3 variant in familial Goodpasture’s and Alport diseases
Source: J Biol Chem. 2021 Mar 26;296:100590. doi: 10.1016/j.jbc.2021.100590 (PMC8100070; doi:10.1016/j.jbc.2021.100590)
Supplement: Supplementary Sections 1–6, Figures S1–S9 and Tables S1, S3 [file mmc2.pdf]

SUPPLEMENTARY APPENDIX

This appendix has been provided by the authors to give readers additional information about their work.

Supplement to: Mechanisms of Collagen IV345 assembly and dysfunction in Goodpasture's and Alport diseases: I. A COL4A3 variant illuminates molecular pathogenesis.

Table of Contents

	Page
**Supplementary Section 1.** ***Clinical histories of two familial Goodpasture's disease patients*** (*Supplementary Fig. 1*)	2
**Supplementary Section 2.** ***Genetic analysis of Zurich variant and proposed mechanism of mutation***	4
**Supplementary Section 3.** ***Prevalence of Zurich variant in sporadic and familial Goodpasture disease and in general population*** (*Supplementary Fig. 2, Supplementary Tables 1 and 2*)	4
**Supplementary Section 4.** ***Pedigree trees of familial GP and Alport patients harboring Z-variant*** (*Supplementary Figs. 3 and 4, Supplementary Table 3*).	6
**Supplementary Section 5.** ***Genotyping of Zurich knock-in mouse*** (*Supplementary Fig. 5*)	9
**Supplementary Section 6.** ***Characterization of homozygous Zurich variant mouse*** (*Supplementary Figs. 6-9*)	9

Supplementary Section 1. Clinical histories of two familial Goodpasture's disease patients.

The index patient. In September 2016, a 24-year-old male (T.A.) presented to the emergency department due to worsening dyspnea and hemoptysis lasting for ten days. In addition, he was febrile and noticed a pink discoloration of his urine. On clinical examination, rales were perceived over both lungs. Laboratory analyses revealed an increased C reactive protein of 47 mg/l (reference < 5 mg/l), leukocytosis of 17.4 G/l (3.0 – 9.6 G/l) and renal impairment with a creatinine of 190 µmol/l (estimated glomerular filtration rate 42 ml/min/1.73m^2). Urinary sediment showed glomerular hematuria with > 10% acanthocytes. Proteinuria in a spot urine sample amounted to 2.5 g/g creatinine, primarily consisting of albumin (2.4 g/g creatinine). Computed tomography of the chest displayed diffuse bi-pulmonary ground glass infiltrates consistent with diffuse alveolar hemorrhage. Immunological tests showed an anti-glomerular basement membrane (GBM) antibody level of 151 U/ml (normal range < 10 U/ml) whereas anti-neutrophil cytoplasmic antibodies (ANCA) and anti-nuclear antibodies (ANA) were negative. The assay (EliA, Pharmacia Diagnostics, Freiburg, Germany) used by immunology laboratory to detect anti-GBM antibodies utilizes the recombinant human α3NC1 domain expressed in insect cells (SF9/baculovirus) as a capture antigen. Because of the typical presentation with pulmonary-renal syndrome and highly positive anti-GBM antibodies a diagnosis of Goodpasture's disease was made.

Additional immunochemical characterization of the serum antibodies of the index patient using NC1 domains and chimeric proteins were performed by indirect ELISA. The results are presented in Fig. ED1A-D. *From the immunochemical analyses, we concluded that antibodies from the index patient (Zurich patient) behaved like antibodies from sporadic GP patients with the reactivity being restricted to the α3NC1 domain of human collagen IV. The Zurich variant did not affect the antigenicity of the dominant GP epitopes and the 8-amino acid Zurich appendage itself did not constitute an epitope.*

Because of rapidly deteriorating pulmonary function, the patient was intubated and mechanically ventilated during the course of the first day of hospitalization. Furthermore, hemodialysis was initiated due to rapidly deteriorating renal function. Therapy with glucocorticoids (three pulses of 1 g methylprednisolone followed by 1mg per kg body weight of oral prednisone per day), plasmapheresis (PEX; 13 sessions within the first 17 days, until anti-GBM antibodies were in the normal range) and cyclophosphamide 1.2 g i.v. was initiated. The patient developed non-occlusive mesenteric ischemia with subsequent peritonitis and severe septic shock. He underwent resection of the terminal ileum and subtotal colectomy with ileostomy and Hartmann's procedure. Because of multiple complications kidney biopsy could not be performed. Owing to the infectious complications, cyclophosphamide was only applied twice with a cumulative dose of 2.4 g. After 8 weeks of treatment in the intensive care unit, he was transferred to a normal ward. Whereas alveolar hemorrhage completely resolved, the patient remained dialysis dependent. He declined to undergo a kidney biopsy at that stage. Corticosteroids were tapered and stopped in October 2018. After having almost normalized following PEX treatment, anti-GBM antibody levels increased again to a maximum of 62 U/ml in July 2017, spontaneously dropped to 5 U/ml in January 2018 and have remained negative since. Currently, the patient is on chronic hemodialysis. Hearing tests excluded sensorineural hearing loss and ophthalmologic examination including retinoscopy was unremarkable.

Index patient's family. The patient's mother (A.A.) underwent renal transplantation at the age of 51 because of renal failure due to Goodpasture's disease. She had presented in 2005 at the age of 45 years with acute dyspnea and hemoptysis lasting for three days. On admission, she had severe renal failure with a serum creatinine of 1739 µmol/l and potassium of 7.2 mmol/l. Urinalysis revealed an active sediment and proteinuria measured in a spot urine sample was 12g/g creatinine. She was positive for anti-GBM antibodies at 72 U/ml, ANA and ANCA were negative.

Bronchoscopy with bronchoalveolar lavage showed diffuse alveolar hemorrhage. Kidney biopsy revealed diffuse crescentic glomerulonephritis with crescents in all 23 glomeruli obtained, eleven cellular, nine fibrocellular and three fibrous. Segmental sclerosis was observed in 6/23 glomeruli. Interstitial fibrosis and tubular atrophy (IFTA) amounted to 30%. Immunofluorescence depicted linear staining for IgG, kappa and lambda light chains (all intensity 3+) and complement C3 (2+) on the GBM. On electron microscopy (EM), the GBM was undulated and partly collapsed but did not show any evidence of thinning, thickening, "basket weaving appearance" or other irregularities. No electron dense deposits were observed (Supplementary Fig. 1). Collagen immunostaining was not performed. On the day of presentation, she was started on hemodialysis. She declined PEX and was treated with three intravenous pulses of methylprednisolone followed by oral prednisone (100 mg per day for a total of ten weeks) and cyclophosphamide. Anti-GBM antibodies normalized three months after initiation of treatment and have remained negative since. Renal function did not recover. After six years of hemodialysis, cadaveric renal transplantation was performed. Six months post-transplant she developed acute T cell mediated rejection with tubulointerstitial inflammation (Banff 1b), which was treated with three methylprednisolone pulses and oral steroids. A second biopsy one month later showed acute T cell mediated vascular rejection with intimal arteritis (Banff IIA). She received five methylprednisolone pulses and was switched from cyclosporine to tacrolimus. Both biopsies did not show evidence for a recurrence of anti-GBM disease in the graft. Currently, the patient has severely decreased graft function with an estimated glomerular filtration rate of 19 ml/min/1.73m^2 according to the CKD-EPI formula. She has a normal hearing capacity and the last ophthalmologic control at the age of 60 years was normal.

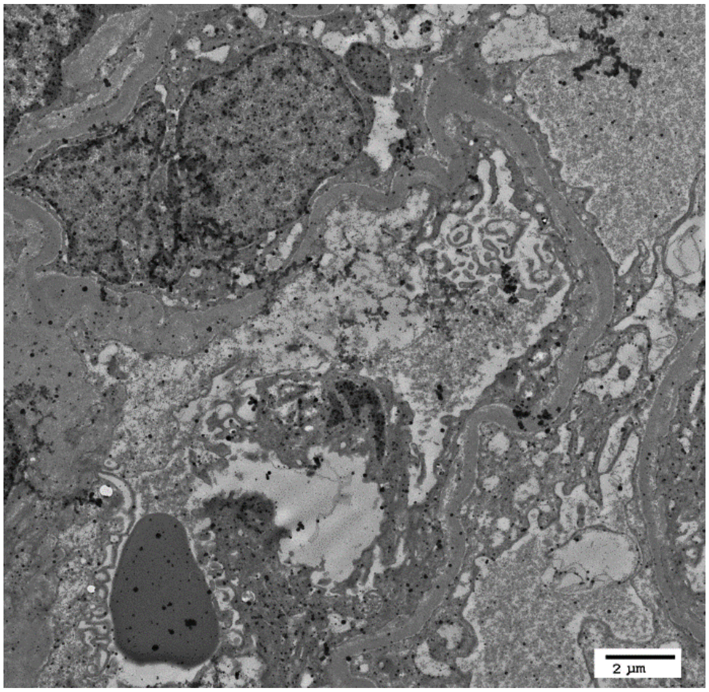

Supplementary Fig. 1. *Electron micrograph of renal tissue from index patient's mother (A.A.).* Electron microscopy showed extensive corrugation with mildly increased *lamina rara interna*, foot process effacement and no lamellation, thinning or basketweaving of the GBM.

The family history of A.A. was negative for Goodpasture's Syndrome, renal failure or alveolar hemorrhage. The mother of A.A. died at the age of 58 reportedly from myocardial infarction, no autopsy was performed. Her father died at the age of 63 after gastric surgery. Autopsy did not show any renal or lung pathology. The brother of A.A. died from an accident. She has two healthy sisters. Besides T.A., A.A. has three other sons. All three are healthy and they have declined any clinical and laboratory investigations so far. The father of T.A. is alive and has no history of kidney disease or hearing loss. Both patients had a positive smoking history (T.A. of 10 pack years and A.A. of 60 pack years at the onset of GP).

Supplementary Section 2. Genetic analysis of Zurich variant and proposed mechanism of mutation

2.1. *Genetic analysis of Zurich variant*
Because we strongly suspected a hereditary cause of the Goodpasture's disease in the two index patients from Zurich, we performed next generation sequencing (NGS) of the genes coding for the α3, α4 and α5 chains of collagen type IV (*COL4A3, COL4A4, COL4A5*) of subjects A.A. and A.T. Genetic analysis revealed a novel heterozygous *COL4A3* mutation c.5010_*del (p.His1670_*167delinsGln*9) in both individuals. This corresponds to an 18-base pair deletion c.5010_*14delCTGAAGCTAAAAAAGACA, including the stop codon, in Exon 52 of the *COL4A3* gene. The deletion is located in the NC1 domain and results in a frameshift mutation and formation of an alternative stop codon (p.His1670_*167delinsGln*9). When translated, the mRNA leads to a structurally aberrant and elongated collagen type IV α3 chain, which lacks the very last amino-terminal amino acid (H) of the wild type protein but instead contains eight additional amino acids (QQNCYFSS). In addition to sequencing of the *COL4A3*, *COL4A4* and *COL4A5* genes, we performed HLA typing of both index patients, because the HLA-DR15*15:01 allele is a known risk factor for GP. The mother (A.A.) is homozygous for the anti-GBM susceptibility HLA-locus DRB1*15:01, whereas the son (T.A.) is heterozygous.

Supplementary Section 3. Prevalence of Zurich variant in sporadic and familial Goodpasture disease and in general population.

3.1. Cohorts of sporadic GP patients
We hypothesized that the Zurich variant might occur at increased frequency in sporadic GP patients. Sporadic GP patients from all analyzed cohorts are listed in Supplementary Table 1 and in a separate supplemental file "Supplementary Table 2 - GP Patients.xlsx".

Supplementary Table 1. *Cohorts of patients with sporadic cases of Goodpasture's disease and clinical criteria used in diagnosis.*

China cohort (n=171):	Anti-GBM disease was defined as positive anti-GBM antibodies in circulation and/or linear immunoglobulin G deposition along the GBM on kidney biopsy (1).
USA cohort (n=35):	Patients were diagnosed with GP disease if they were positive for 1) glomerular crescents and 2) linear anti-GBM staining<br>This study examined whole-exome data available from a Vanderbilt cohort of GP patients.
UK cohort (n=29)	22 patients had renal biopsy proven anti-GBM disease (of which 20 had circulating anti-GBM antibodies) and 7 were diagnosed on clinical findings and positive anti-GBM serology alone.
Sweden cohort (n=15):	1) circulating anti-GBM antibodies, 2) clinical history of rapidly progressive glomerulonephritis (2).
Swiss cohort (n=11):	10 out of 11 GP patients were diagnosed with the disease based on the results of their serology tests (circulating anti-GBM antibody) as well as by biopsy results (linear staining and diffuse (9 patients) or focal (1 patient) extracapillary proliferation); one patient was diagnosed by serology results in combination with the clinical picture.

Supplementary Table 2. *A list of Goodpasture's disease patients (see a separate supplemental file "Supplementary Table 2 - GP Patients.xlsx").*

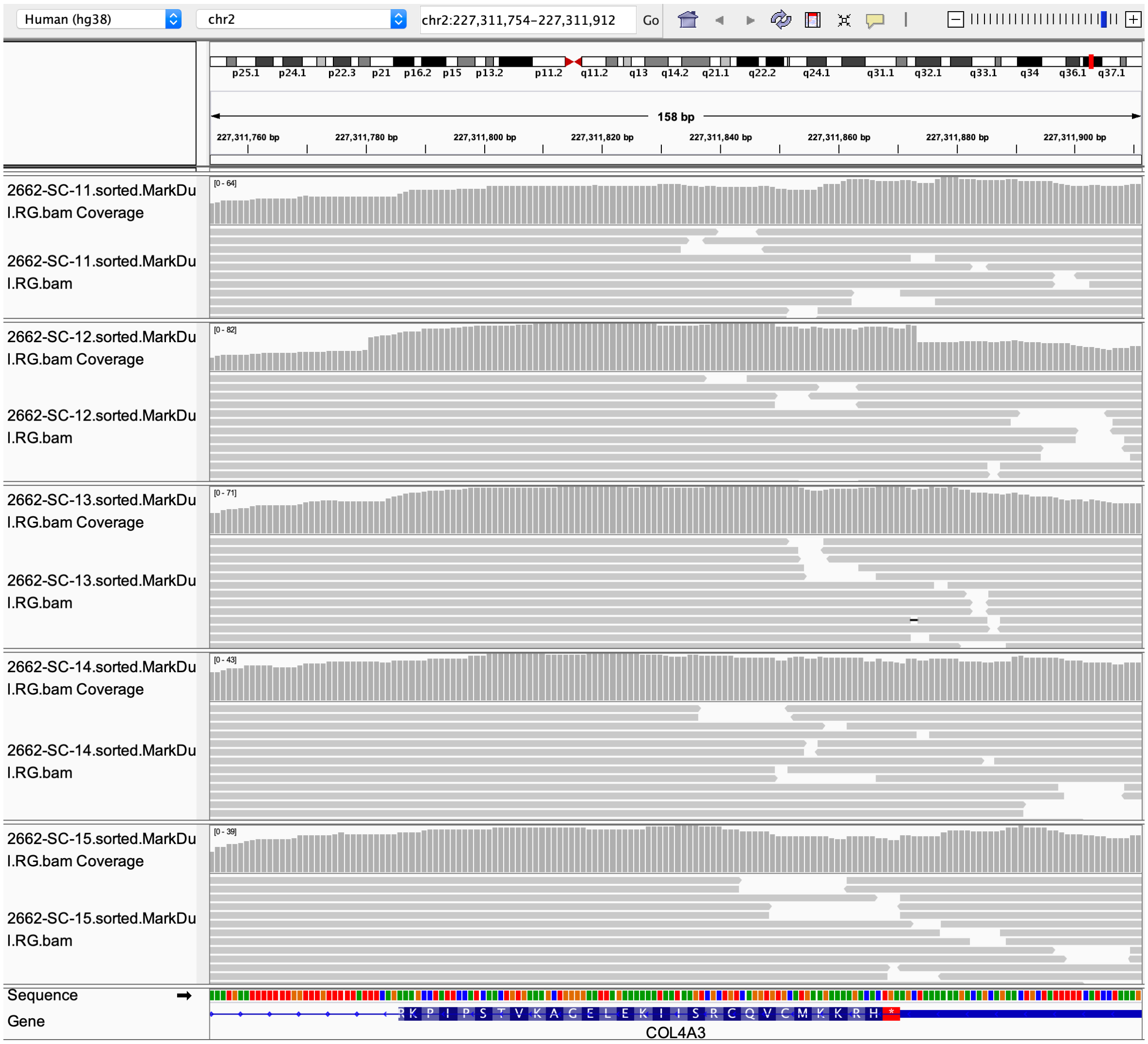

Supplementary Fig. 2. *Representative WES results of sporadic GP patients from USA cohort (patients 11 through 15) visualized with IGV browser.* The results show exon 52 of COL4A3 gene and demonstrate that there were no alterations from reference sequence in this genomic region.

All patients with rapidly progressive glomerulonephritis have been diagnosed with Goodpasture's disease based on the results of their histology and/or serology in combination with clinical data. We completed whole-exome or whole-genome sequencing in 261 sporadic Goodpasture's disease (GP) patients from five cohorts (Supplementary Table 1). All targeted bases (*COL4A3* gene exon 52) in WES experiments show at least 20 independent reads. The average sequencing depth for all bases in targeted region in WGS experiments was at least x50. Representative WES results of sporadic GP patients (USA cohort) show exon 52 of *COL4A3* gene and demonstrates that there were no alterations from reference sequence in this genomic region (Supplementary Fig. 2). *We found no evidence of rs765655100 deletion/insertion and concluded that there was no evidence of Zurich variant in the studied cohort of sporadic Goodpasture's disease.*

3.2. Other familial GP cases

Reported cases of familial GP disease are extremely rare (3). In one recently reported family, HLA-typing of two cases revealed heterozygosity for HLA-DRB1*15 (HLA-DR15 serotype), while sequencing of *COL4A3*, *COL4A4* and *COL4A5* had not been performed in this kindred (3). We therefore obtained genomic DNA from an affected patient of the reported family and performed sequencing of *COL4A3*, *COL4A4* and *COL4A5*, which *did not show any pathogenic variant*. One patient harbored the benign *COL4A3* variant c.346C>A p.Pro116Th (minor allele frequency 0.8%). *Thus, Zurich variant does not occur in all cases of familial GP disease.*

3.3. General population of unknown phenotype

According to the Genome Aggregation Database (gnomAD), the allele frequency of Zurich variant in the general population is 0.00006416 (*16* cases out of 249376 alleles) with a frequency of 0.0001325 (16/113172) in the non-Finnish European population and no cases in other populations (http://gnomad.broadinstitute.org/variant/2-228176574-GAAAAGACACTGAAGCTAA-G, as of May 21, 2020). A search of the NCBI ClinVar database for rs765655100 further revealed individuals bearing the variant reported by Myriad Women's Health (formerly Counsyl). Overall, the variant was identified in *32* individuals out of ca. 200000 alleles tested by a carrier screening test that includes >200 genes potentially associated with hereditary diseases. Clinical details on these individuals are unavailable, as are the indications for screening, but presumably, the majority of individuals being tested are healthy (personal communication from Megan Judkins, Myriad Women's Health).

Supplementary Section 4. Pedigree trees of familial GP and Alport patients harboring Z-variant.

Pedigree trees of familial GP disease and four families with Alport syndrome (AS) harboring Zurich variant are shown in Supplementary Fig.3. Clinical and genetic data for the four AS families are summarized in Supplementary Table 3. Representative sequencing results are shown in Supplementary Fig. 4.

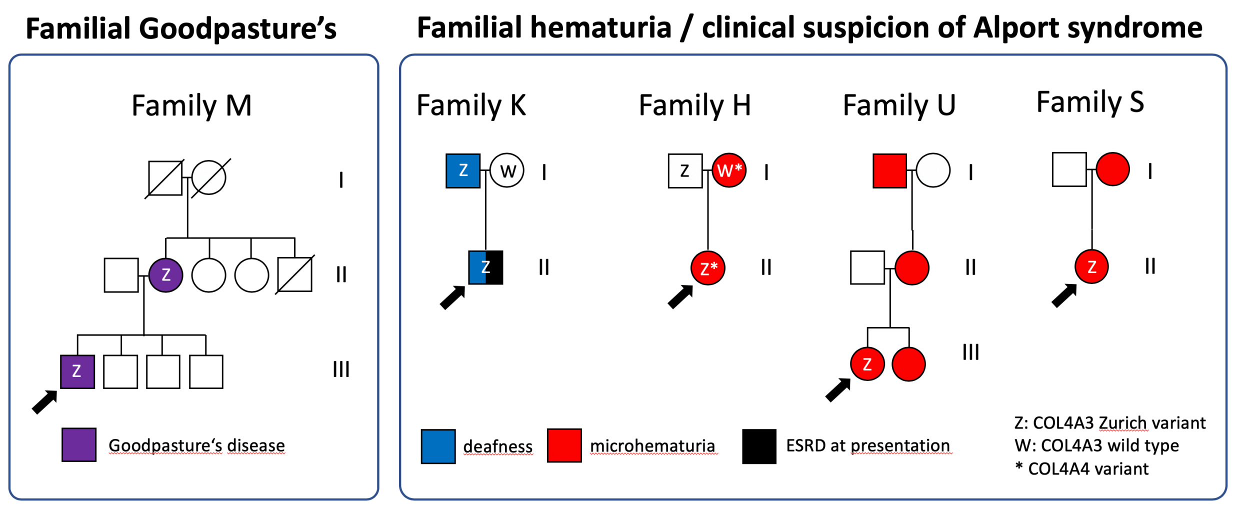

Supplementary Fig. 3. *Pedigree trees of familial GP disease and four families with Alport syndrome harboring Zurich variant.* The arrows denote index patients, clinical phenotypes are indicated by colors, circles denote female patients, squares denote male patients deceased individuals are crossed. Roman numerals denote generations. In genetically tested individuals, the genotypes are indicated within the symbols (Z, COL4A3 rs765655100; W, COL4A3 wild type; *, COL4A4 variant c.1321_1369+3del).

	Family K			Family H			Family U				Family S	
Number of individuals with clinical data	3			3			4				2	
Patient ID	J5412/16	J1491/17	J1493/17	J5397/17	J5462/18	J5463/18	J8435/18				J5271/19	
Origin	Greek			Arabic			Mother Caucasian, father Turkish ethnicity				Turkish	
Gender	M	F	M	F	F	M	F	F	F	M	F	F
Relation to index patient	index	mother	father	index	mother	father	index	sister	mother	mat gf	index	mother
Indication for genetic testing	ESRD and deafness			familial hematuria			familial hematuria				familial hematuria	
DOB	1993		1960	2009			2012				1992	
Age at presentation	23			6			5				24	
Hearing problems	sensory deafness	no	deafness	no			no	no	no	no	no	
Heaematuria	no		no	mh	mh	no	mh	mh	mh	mh	mh	mh
eGFR, ml/min/1.73 m2	on dialysis	>90	95	104	>90	>90	175	reportedly normal			143	
serum creatinine (mg/dl)	on dialysis		0.9	0.42			37				0.4	
Proteinuria , g/day or g/g Crea	1.9		0.064	<0.15	<0.15	<0.15	0.086				0.26	
albumin, mg/g Crea				<5			<20				7	
Other renal disease					urolithiasis	urolithiasis						
**Genetics**												
Zurich variant (COL4A3 rs765655100)	**YES**	WT	**YES**	**YES**	WT	**YES**	**YES**	-	-	-	**YES**	-
COL4A4: c.1321_1369+3del52				YES	YES							

Supplementary Table 3. *Clinical and genetic data for Alport families with Zurich variant.*

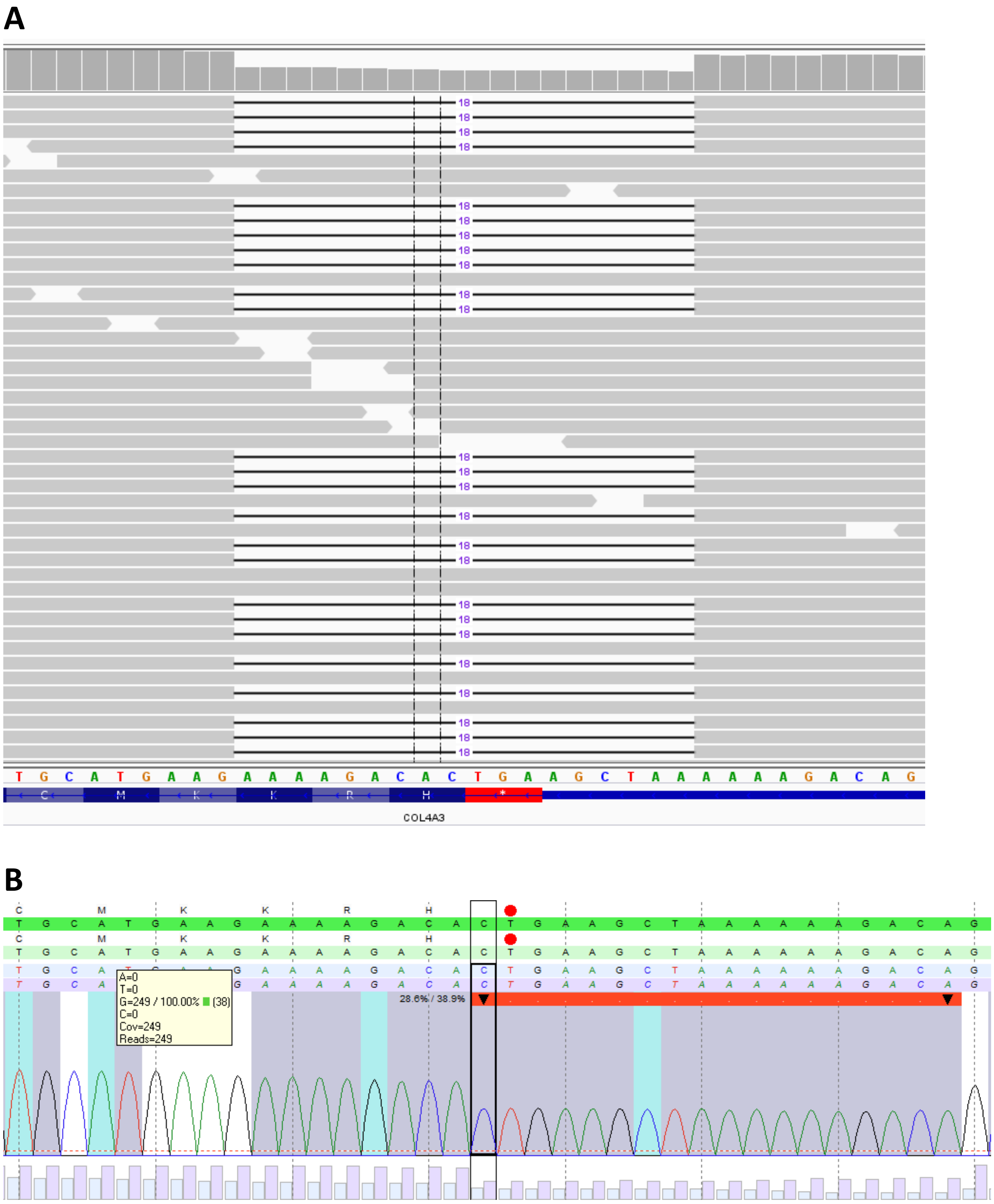

Supplementary Fig. 4. *Representative sequencing results of patients with familial microhematuria tested positive for the Zurich variant with the Alport NGS panel*. A) NGS visualized with IGV browser; B) Sanger sequencing.

Supplementary Section 5. Genotyping of Zurich knock-in mouse.

Two different schemes were used to genotype Zurich variant knock-in mouse model:
1) The following primers, sense TGCAGGAAAGCCTATGTTCTATG and anti-sense AGTGAAACTTTGCACTACTACATC, were used to generate PCR products of 460 bp in wild type and 446 bp in the mutant. Mutant PCR product had NsiI restriction site, while wild type did not. PCR products were digested with NsiI enzyme resulting in two bands for the mutant (main text Fig. 3B).
2) The following primers were used: specific to mutant NFMUTS1: 5'- TGA AGA AAA GAC AGC AGA ACT GCT A -3'; specific to wild type NFWTS1: 5'-AGG TGT GCA TGA AGA AAA GAC ATT GAC GAA ATC-3'; common for mutant and wild type alleles NRMUTWTS1: 5'- GTG CAC ATT TAG TAA GCA GTG TCC -3'. Two PCR reactions were performed. NFMUTS1 and NRMUTWTS1 primers were used to identify presence of the mutant allele. The PCR amplification resulted is an 827 bp product. NFWTS1 and NRMUTWTS1 primers were used to identify presence of the wild type allele, resulting in production of an 836 bp fragment (Supplementary Fig. 5).

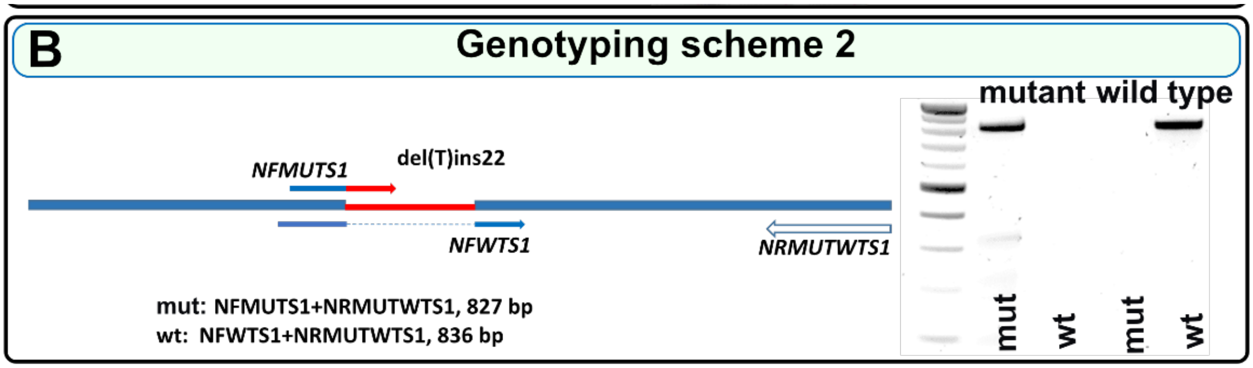

Supplementary Fig. 5. *Genotyping scheme 2.*

Supplementary Section 6. Characterization of homozygous Zurich variant mouse.

6.1. Renal phenotype

To determine if the collagen $IV^{\alpha345}$ scaffold is present in the GBM of the Zurich variant knock-in (*Col4a3*$^{z/z}$) mice, we performed immunofluorescent staining of kidney sections with antibodies specific to α3, α4 and α5 chains (Supplementary Fig. 6). The results demonstrate linear staining of similar intensity in wild type control and homozygous *Col4a3*$^{z/z}$ for all three chains (Supplementary Fig. 6). As expected, no staining was observed in the glomerulus of *Col4a3*$^{-/-}$ mouse as has been previously shown(4). Therefore, the variant α3 chain of collagen IV is deposited in the GBM, and the deposition of α4 and α5 chains is unaffected in *Col4a3*$^{z/z}$ mouse. *This indicates that the α3 chain bearing Z-appendage incorporates into collagen* $IV^{\alpha345}$ *scaffold.*

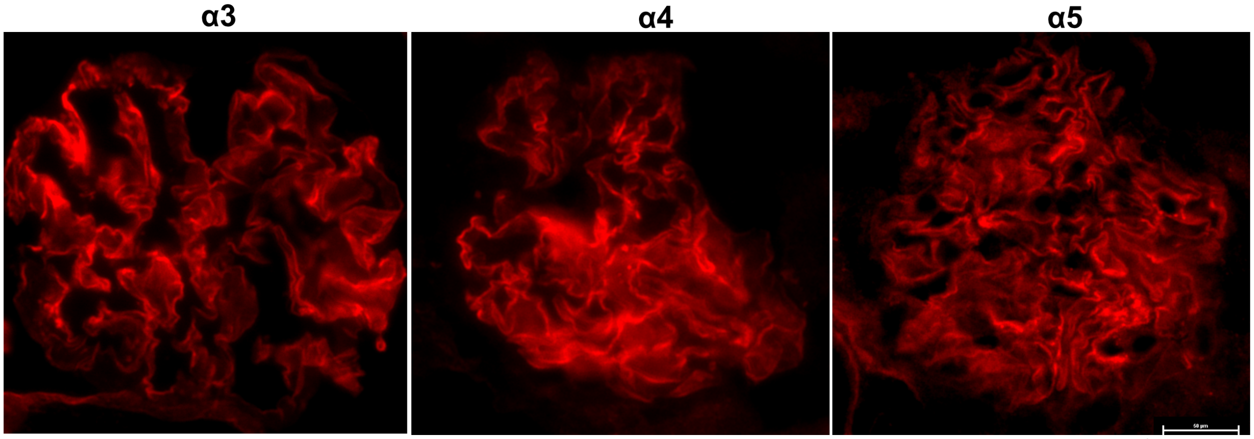

Supplementary Fig. 6. *Deposition of α3, α4 and α5 chains of collagen IV in the GBM of Col4a3$^{z/z}$ mouse.* The mouse was 16 weeks of age and developed severe albuminuria (ACR=1500mg/g).

The NC1 domain is the key protein modulator of collagen IV scaffolds and GBM assembly. It plays central role in the supra-structural organization of collagen IV scaffolds via protomers oligomerization outside the cell. Inability or compromised ability of the α345 collagen IV protomers to associate via their NC1 domains will trigger a sequence of events leading to assembly of a defective collagen scaffolds and GBM. To determine whether the *Col4a3*$^{z/z}$ variant harboring an 8-residue extension of α3 chain called the Z-appendage, interferes with the ability of α345 collagen IV protomers to form hexamers we performed Western blot analysis with chain specific antibodies. Kidneys from wild type control and Col4a3$^{z/z}$ mice were homogenized and NC1 domains of collagen IV were excised and solubilized using collagenase digestion. All three NC1 domains, α3, α4 and α5, were equally present in monomeric and cross-linked dimeric forms in the control and *Col4a3*$^{z/z}$ kidney (main text Fig. 4), indicating that the α345 scaffold with the Z-appendage is fully incorporated into the GBM.

These results confirm the ability of α345 trimeric protomers, bearing the Z-appendage to form cross-linked hexamers. *Thus, in kidneys, Zurich variant does not interfere with association of α345 collagen IV protomers into hexamers followed by formation of sulfilimine crosslinks. Furthermore, the results indicate that the Z-appendage is a reporter group on the α345 hexamer that can shed light on function and pathogenic mechanism.*

We initially determined the albumin-to-creatinine ratio (ACR) in the spot urine samples from a group of six *Col4a3*$^{z/z}$ and five wild type control mice. The measurements were performed once a week starting from week 9. Moderate albuminuria (average ACR of 100 mg/g) was observed in all *Col4a3*$^{z/z}$ animals at week 9 following by increase with substantial variation among the mice. Individual ACR plots for each analyzed mouse are presented in Supplementary Fig. 7.

Overall, we monitored albuminuria in nine *Col4a3*$^{z/z}$ mice (including three that were later used for EM analysis). A majority of mice were moderately affected with ACR between 100 and 400 mg/g (Supplementary Fig. 7). However, two out of nine mice demonstrated a dramatic (~10-fold) increase in albuminuria by 14-16 weeks of age (Supplementary Fig. 8).

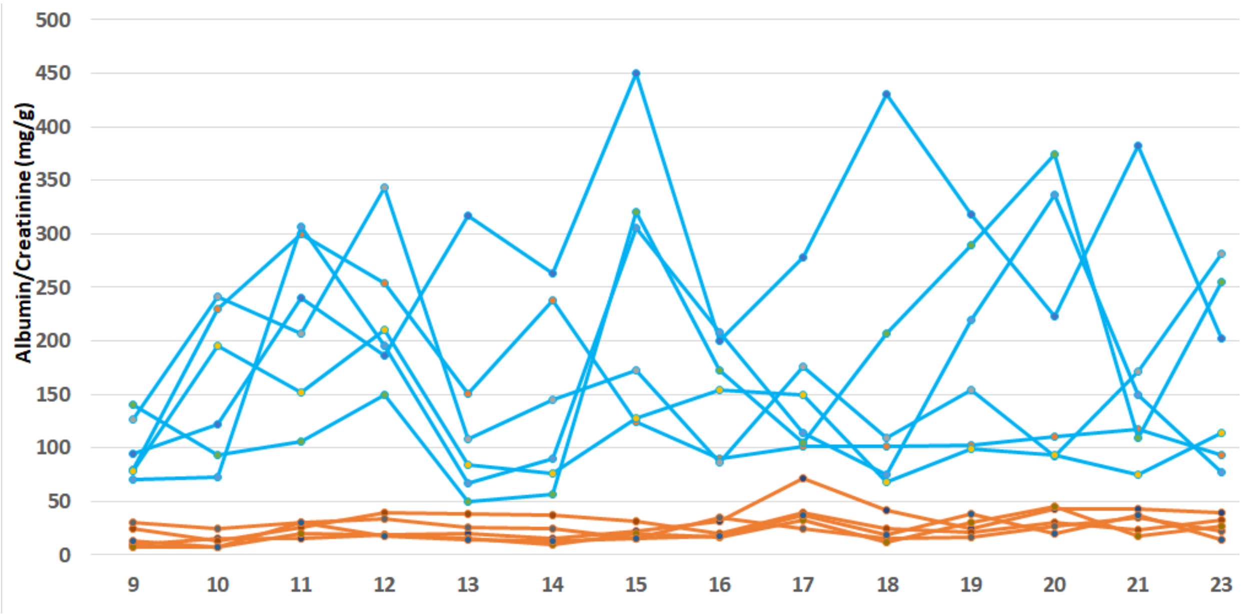

Supplementary Fig. 7. *Urinary albumin-to-creatinine ratios of WT and Col4a3$^{z/z}$ mice.* The ratios are plotted for each individual mouse from 9 to 23 weeks of age. Six Col4a3$^{z/z}$ and five wild type controls were measured. Blue lines represent data from Col4a3$^{z/z}$ mice, orange lines represent data from WT controls.

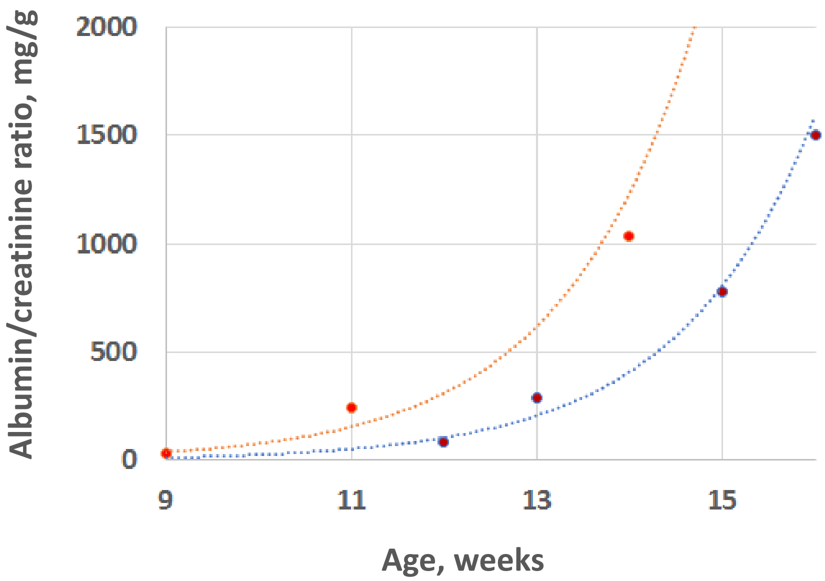

Supplementary Fig. 8. *Albumin-to-creatinine ratios in two Col4a3$^{z/z}$ mice with the most severe albuminuria.*

In conclusion, incorporation of the α3 chain bearing Z-appendage into collagen IV$^{\alpha345}$ scaffold of the GBM caused glomerulosclerosis and albuminuria.

6.2. Circulating antibody

To check if Col4a3$^{z/z}$ mutation resulting in developing of circulating antibody targeting α3NC1 monomer, a major target for Goodpasture autoantibody in human, we analyzed serum from Zurich

knock-in and wild type mice by indirect ELISA. No significant binding to α3NC1 monomer above the background or negative control (α1NC1) was found for both groups (Supplementary Fig. 9). With established assay sensitivity of less than 1 ng for Mab3 antibody, *this indicates the absence of circulating α3-specific antibody in Zurich mouse.*

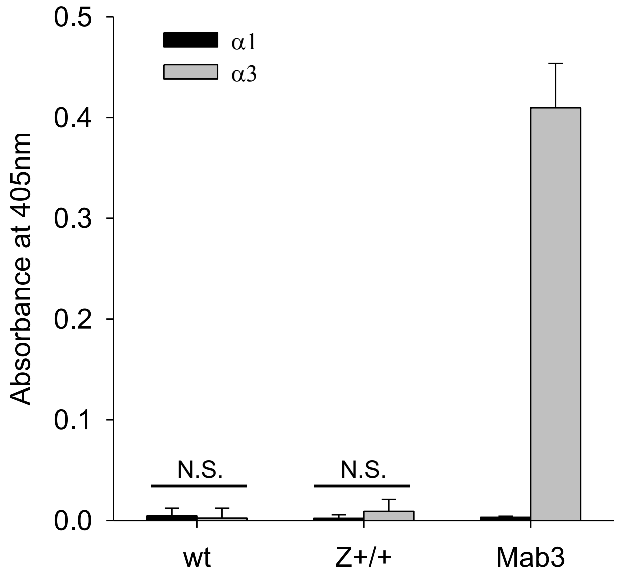

Supplementary Fig. 9. *Indirect ELISA for the presence of α3-specific antibody in serum of WT and Col4a3$^{z/z}$ (Z+/+) homozygous mice.* Plate was coated with α3NC1 (*grey bars*) and α1NC1 (*black bars*), which was used as a negative control. All sera were tested at 1:100 dilution. Low concentration of mouse monoclonal antibody Mab3 (3 ng/well) was used as a positive control. No significant biding (N.S.) was detected in both groups above the background. Bars represent average values ± SD for six animals per group.

Supplementary References

1. Cui, Z., Zhao, J., Jia, X. Y., Zhu, S. N., and Zhao, M. H. (2011) Clinical features and outcomes of anti-glomerular basement membrane disease in older patients. *Am J Kidney Dis* **57**, 575-582
2. Persson, U., Hertz, J. M., Carlsson, M., Hellmark, T., Juncker, I., Wieslander, J., and Segelmark, M. (2004) Patients with Goodpasture's disease have two normal COL4A3 alleles encoding the NC1 domain of the type IV collagen alpha 3 chain. *Nephrol Dial Transplant* **19**, 2030-2035
3. Angioi, A., Cheungpasitporn, W., Sethi, S., De Vriese, A. S., Lepori, N., Schwab, T. R., and Fervenza, F. C. (2017) Familial antiglomerular basement membrane disease in zero human leukocyte antigen mismatch siblings. *Clin Nephrol* **88**, 277-283
4. Cosgrove, D., Meehan, D. T., Grunkemeyer, J. A., Kornak, J. M., Sayers, R., Hunter, W. J., and Samuelson, G. C. (1996) Collagen COL4A3 knockout: a mouse model for autosomal Alport syndrome. *Genes & development* **10**, 2981-2992
